# Senescence plays a role in myotonic dystrophy type 1

**DOI:** 10.1172/jci.insight.159357

**Published:** 2022-10-10

**Authors:** Mikel García-Puga, Ander Saenz-Antoñanzas, Gorka Gerenu, Alex Arrieta-Legorburu, Roberto Fernández-Torrón, Miren Zulaica, Amets Saenz, Joseba Elizazu, Gisela Nogales-Gadea, Shahinaz M. Gadalla, Marcos J. Araúzo-Bravo, Adolfo López de Munain, Ander Matheu

**Affiliations:** 1 Cellular Oncology Group and; 2 Neuroscience Area, Biodonostia Health Research Institute, San Sebastian, Spain.; 3 IKERBASQUE, Basque Foundation for Science, Bilbao, Spain.; 4 Neuromuscular and Neuropediatric Research Group, Germans Trias i Pujol, Health Research Institute, Badalona, Spain.; 5 Clinical Genetics Branch, National Cancer Institute, NIH, Bethesda, Maryland, USA.; 6 Computational Biology and Systems Biomedicine Group, Biodonostia Health Research Institute, San Sebastian, Spain.; 7 Centro de Investigación Biomédica en Red de Fragilidad y Envejecimiento (CIBERfes), Carlos III Institute, Madrid, Spain.; 8 Neurology Department. Donostia University Hospital, OSAKIDETZA, San Sebastian, Spain.; 9 Centro de Investigación Biomédica en Red de Enfermedades neurodegenerativas (CIBERNED), Carlos III Institute, Madrid, Spain.; 10 Faculty of Medicine and Nursery, Department of Neurosciences, University of the Basque Country, San Sebastian, Spain.

**Keywords:** Aging, Cell Biology, Cellular senescence, Drug therapy, Neuromuscular disease

## Abstract

Myotonic dystrophy type 1 (DM1; MIM #160900) is an autosomal dominant disorder, clinically characterized by progressive muscular weakness and multisystem degeneration. The broad phenotypes observed in patients with DM1 resemble the appearance of an accelerated aging process. However, the molecular mechanisms underlying these phenotypes remain largely unknown. Transcriptomic analysis of fibroblasts derived from patients with DM1 and healthy individuals revealed a decrease in cell cycle activity, cell division, and DNA damage response in DM1, all of which related to the accumulation of cellular senescence. The data from transcriptome analyses were corroborated in human myoblasts and blood samples, as well as in mouse and *Drosophila* models of the disease. Serial passage studies in vitro confirmed the accelerated increase in senescence and the acquisition of a senescence-associated secretory phenotype in DM1 fibroblasts, whereas the DM1 *Drosophila* model showed reduced longevity and impaired locomotor activity. Moreover, functional studies highlighted the impact of BMI1 and downstream p16^INK4A^/RB and ARF/p53/p21^CIP^ pathways in DM1-associated cellular phenotypes. Importantly, treatment with the senolytic compounds Quercetin, Dasatinib, or Navitoclax reversed the accelerated aging phenotypes in both DM1 fibroblasts in vitro and in *Drosophila* in vivo. Our results identify the accumulation of senescence as part of DM1 pathophysiology and, therefore, demonstrate the efficacy of senolytic compounds in the preclinical setting.

## Graphical abstract

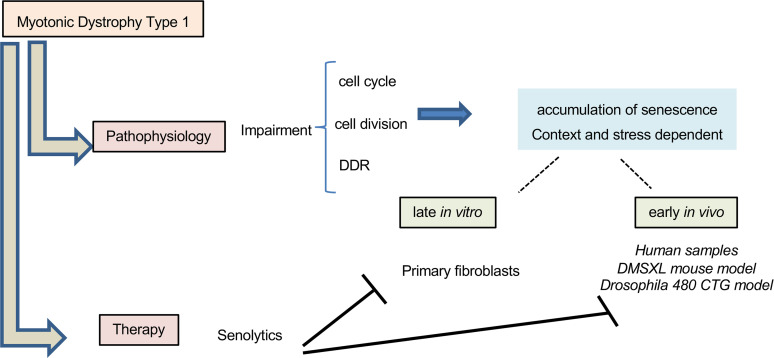

## Introduction

Myotonic dystrophy (DM) is an autosomal dominant multisystem form of muscular dystrophy, with an estimated incidence of 1 in 8,000 people worldwide (1). Based on genetic alterations, DM is clinically classified in 2 distinct forms. DM type 1 (DM1; MIM #160900), also known as Steinert’s disease, is caused by the accumulation of cytosine-thymine-guanine (CTG) trinucleotide repeat expansions in the 3′ untranslated region of the dystrophia myotonic-protein kinase (*DMPK*) gene on chromosome 19q13.3 (2). DM type 2 (DM2; MIM #602668) results from a tetranucleotide CCTG expansion in the zinc finger protein 9 (*ZNF9*) gene on chromosome 3q21.3 (3). At the molecular level, when these genes are transcribed, pathogenic transcripts are generated, and these are retained in the nuclei of cells as ribonuclear aggregates. These aggregates interfere with and disrupt RNA-binding proteins that regulate alternative splicing. The accumulation of RNA toxicity plays a major role in the pathology of the disease, due to microsatellite expansion in noncoding regions that can be transcribed into pathogenic transcripts. This alters alternative splicing processes, modifies the bioavailability of splicing factors, and increases the number of fetal mRNA isoforms. Consequently, there are deregulated patterns of protein expression, with negative effects on multiple tissues, thus contributing to the multisystem pathogenesis of DM (1, 4).

DM1 is more common than DM2 and represents a more severe phenotype (5). In DM1, mutant *DMPK* transcripts accumulate in ribonuclear foci that interfere with 2 families of proteins that regulate alternative splicing, muscleblind-like (MBNL) and CUGBP Elav-like family. The MBNL family comprises MBNL1 and MBNL2 (jointly denoted MBNL1/2), which show overlapping patterns of expression in skeletal muscle, heart, and the CNS and have partially redundant functions. In these tissues, the function of MBNL1/2 is reduced, while CUGBP1 is activated, by aberrant binding to CTG expansions. These changes maintain the fetal alternative splicing patterns of transcripts in adults, resulting in specific symptoms (1, 4). Importantly, the length of the CTG expansion increases with each generation, and this is associated with an earlier age of onset of the disease and a more severe phenotype (6). Thus, unaffected individuals carry less than 50 triplet repeats, whereas expansions ranging between 50 and 4,000 CTG repeats are present in affected individuals (6).

Patients with DM1 present a multisystem degenerative process that includes progressive muscular weakness and atrophy, myotonia, cardiomyopathy, insulin resistance, cataracts, increased cancer incidence, dementia-related Tau protein deposits in the brain, metabolic syndrome, and premature death. Respiratory distress and heart failure are the most frequent causes of death (7, 8). Different pathogenic mechanisms, including alteration of autophagy (9–11), mitochondrial dysfunction (10, 12, 13), senescence accumulation and telomere shortening (14–18), and genomic instability (19) have been associated with DM1 phenotypes. However, the detailed experimental validation of these mechanisms remains incomplete, and the extent to which each mechanism contributes to the development of the disease has not yet been clearly defined.

In order to understand the underlying pathogenic mechanism of DM1, we performed a transcriptomic analysis of primary fibroblasts derived from healthy donors and patients with DM1. We found that DM1 cells showed impaired cell cycle progression, cell division, and DNA damage response (DDR), as well as increased accumulation of senescent cells. These results were validated in patient-derived myoblasts and peripheral blood mononuclear cells (PBMCs), as well as in *DMSXL* mouse (20) and *REC2* *Drosophila* (21) models of the disease. Remarkably, treatment of human fibroblasts and myoblasts, as well as the *Drosophila* model with senolytic compounds (Quercetin, Dasatinib, and Navitoclax), resulted in the recovery of multiple DM1-related phenotypes and may represent a potential therapy for these patients.

## Results

### DM1-derived fibroblasts resemble molecular characteristics of DM1 patient pathophysiology.

**Figure 1:**
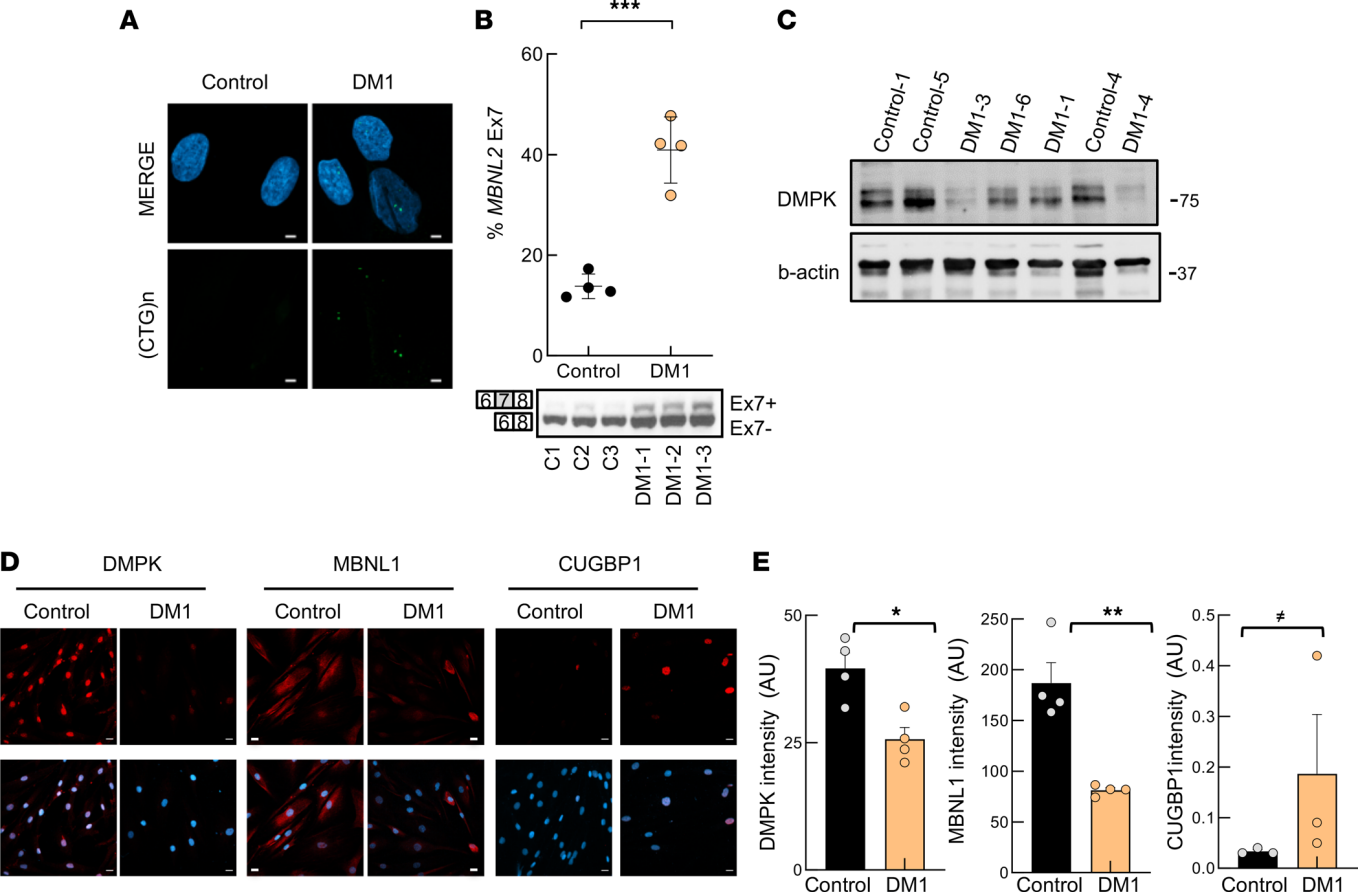
The molecular characteristics of DM1-derived fibroblasts resemble those of DM1 patient pathophysiology. (**A**) Representative fluorescence of FISH images showing (CTG)n foci in DM1 and control fibroblasts (*n* = 4). Scale bar: 5 μm. (**B**) RT-PCR and representative immunoblot of alternative splicing changes of the *MBNL2* transcript in DM1 and control fibroblasts (*n* = 4). Upper band represents the exon inclusion isoform (Ex7+) and lower band represents the exon exclusion isoform (Ex7–). (**C**) Representative immunoblot of DMPK protein levels in fibroblasts derived from patients with DM1 and controls stained with Santa Cruz antibody (sc-134319). (**D** and **E**) Representative images and quantification of DMPK, MBNL1, and CUGBP1 immunofluorescence in fibroblasts derived from patients with DM1 and controls (*n* = 3) Scale bar: 20 μm. Each point represents 1 independent fibroblast culture. *P* values were calculated using the Student’s *t* test. ^≠^*P* < 0.1, *P < 0.05, **P < 0.01, ***P < 0.001.

Fibroblasts constitute a well-established model for the study of cell aging in vitro (22). Since DM1 is a multisystem disease that resembles accelerated aging (17, 23), we established and characterized fibroblast cultures from 7 different patients with DM1 and 6 healthy donors (Supplemental Table 1; supplemental material available online with this article; https://doi.org/10.1172/jci.insight.159357DS1). First, we determined that fibroblasts recapitulated molecular alterations observed in patients with DM1. We detected the accumulation of nuclear foci (Figure 1A) and the expression of the neonatal isoform of *MBNL2*, with the inclusion of exon 7, in DM1 fibroblasts (Figure 1B). Moreover, we studied the expression of DMPK, MBNL1, and CUGBP1 in DM1 fibroblasts. Western blot and immunofluorescence revealed that DMPK expression was reduced at the protein level (Figure 1, C–E, and Supplemental Figure 1, A and B). The lower levels of DMPK are in agreement with previous studies in vitro and in vivo (24–26). Immunofluorescence showed the presence of the characteristic MBNL1 nuclear foci but also an overall decrease in MBNL1 levels in DM1-derived fibroblasts (Figure 1, D and E). On the contrary, CUGBP1 was elevated (Figure 1, D and E). Finally, we measured the number of CTG expansions in patient blood samples during the diagnosis of the disease and in the fibroblasts in culture. We detected some differences between blood and primary fibroblasts, where the number of CTG expansions increased under serial passage in culture (Supplemental Table 1). The expression of DMPK remained reduced in DM1 fibroblasts under serial passage conditions (Supplemental Figure 1C). These results indicate that patient-derived fibroblasts recapitulate some molecular and genetic characteristics of patients with DM1.

### Transcriptomic study reveals multiple processes differentially altered in DM1 fibroblasts.

**Figure 2:**
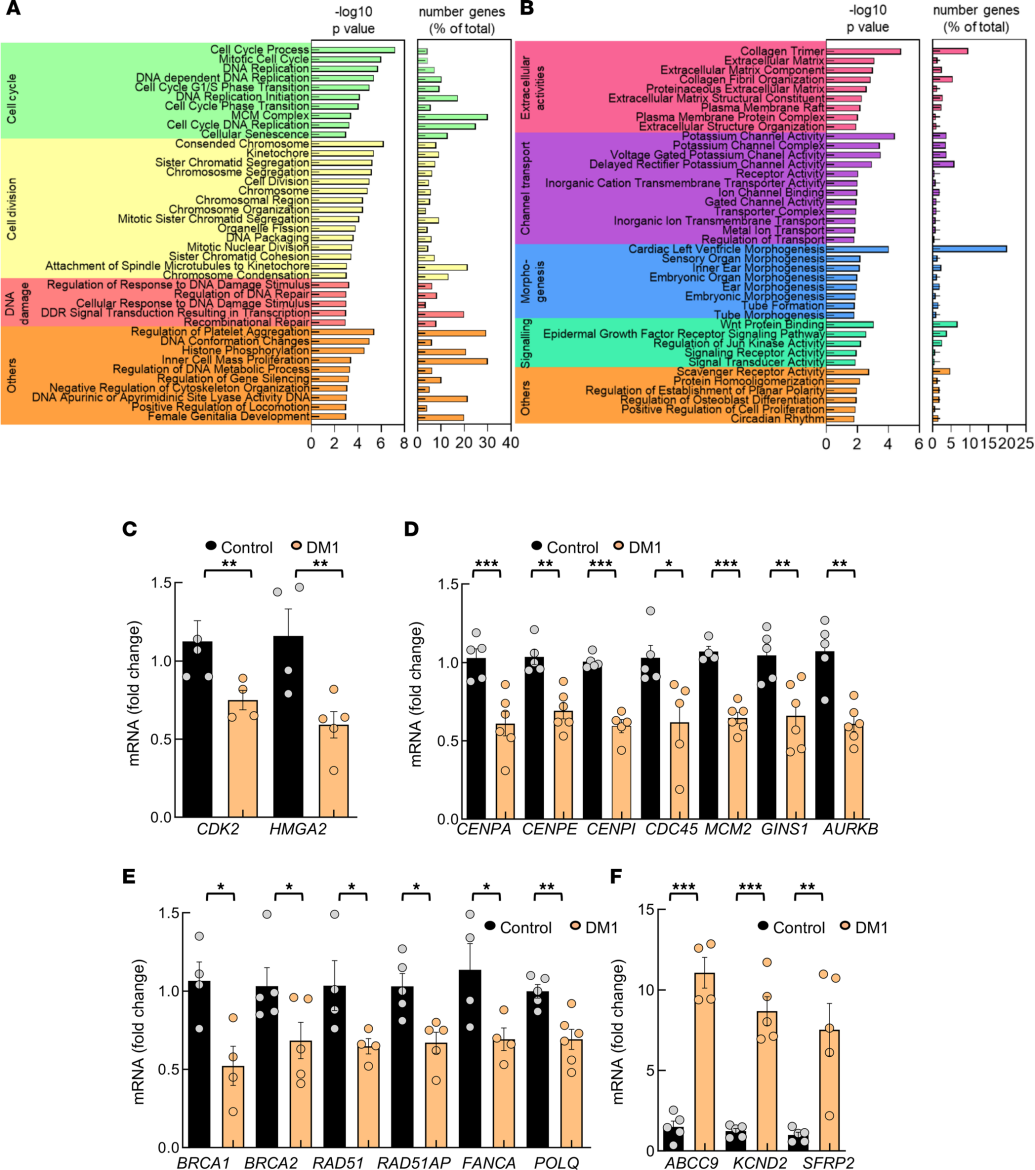
Transcriptomic studies reveal transcripts associated with multiple processes differentially expressed in DM1 fibroblasts. (**A** and **B**) Bar plot of the –log_10_(*P*) and percentage of genes altered in each term of the significantly downregulated (**A**) and upregulated (**B**) terms in DM1 fibroblasts (*n* = 5) compared with controls (*n* = 3). Longer bars correspond to greater statistical significance of the enrichment. The significance of the gene set enrichment (*P* value) was calculated using the hypergeometric distribution. The multitest effect influence was corrected using the Benjamini-Hochberg correction test. (**C**–**F**) qPCR analysis of the indicated genes related to cell cycle (**C**), cell division (**D**), DNA damage repair (**E**), and extracellular matrix and potassium pathways (**F**) in DM1 and control fibroblasts (each point represents an independent sample). *P* values were calculated using the Student’s *t* test. *P < 0.05, **P < 0.01, ***P < 0.001.

We performed transcriptomic studies in DM1 and control fibroblasts. We identified 378 genes that were significantly altered in DM1 fibroblasts, including 292 downregulated and 86 upregulated transcripts (Supplemental Tables 2 and 3). Gene Ontology (GO) enrichment analysis showed that cell cycle, cell division and replication, and DDR were the processes most significantly altered in DM1 derived fibroblasts, all of which were downregulated (Figure 2, A and B). Within the cell cycle–associated genes, there were genes involved in phase transition such as *CDK2*, *CDK6* (27), and *FOXM1* (28); regulators of cyclin-dependent kinase (CDK) inhibitors, such as *HMGA2* (29); and proliferation markers, such as *Ki67*. We validated by quantitative PCR (qPCR) the lower levels of *CDK2* and *HMGA2* in DM1 fibroblasts (Figure 2C). Among cell division, decreased expression of genes involved in DNA replication such as *CDC45*, *MCM3*, *MCM4*, *MCM2-7*, and *GINS1* (30, 31); genes required to preserve the centromere position, such as *CENPA* (32); members of the constitutive centromere-associated network, such as *CENPE*, *CENPI*, *CENPM*, and *CENPW* (33); regulators of DNA replication and repair, like *TRAIP* (34); and genes involved in chromosome condensation and mitosis, such as *AURKB* (35) were detected. qPCR confirmed the lower levels of *CENPA*, *CENPE*, *CENPI*, *CDC45*, *MCM2*, *GINS1*, and *AURKB* in DM1 fibroblasts (Figure 2D). The expression of several DDR genes was also reduced, including *BRCA1*, *BRCA2*, *RAD51*, *RAD51AP*, *FANCA*, and *POLQ*, which were validated by qPCR (Figure 2E). On the contrary, processes involving extracellular matrix and potassium-related pathways were elevated in DM1 fibroblasts. Among them, the increased levels of *ABCC9*, *KCND2*, and *SFRP2* expression were confirmed by qPCR in DM1 cells (Figure 2F). Taken together, our results reveal that critical processes for cell homeostasis, such as the cell cycle, cell division, DNA replication, and DDR are deregulated in DM1 cells.

### In vivo validation in DM1-derived blood and muscle samples.

**Figure 3:**
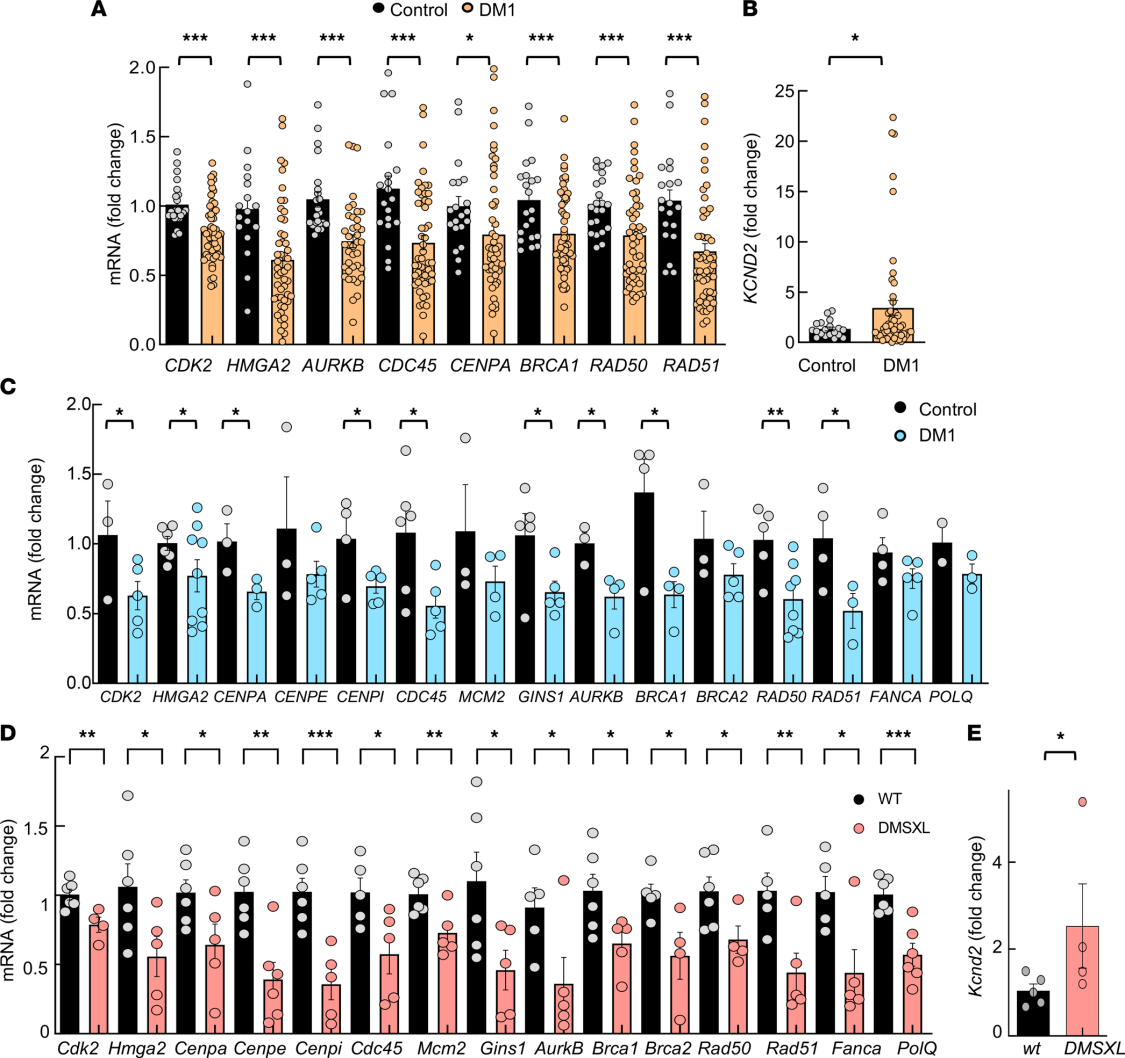
Transcriptomic findings are extended to additional DM1 models in vitro and in vivo. (**A**) qPCR analysis of the indicated genes in PBMCs from DM1 and controls in PBMCs derived from patients with DM1 (*n* ≥ 56) and controls (*n* ≥ 22) (each point represents an independent sample). (**B**) qPCR analysis of *KCND2* in PBMCs. (**C**) Measurement of mRNA levels of indicated genes by qPCR in early-passage myoblasts (*n* ≤ 7) derived from patients with DM1 and controls (each point represents an independent experiment). (**D** and **E**) Measurement of mRNA levels of indicated genes by qPCR in tibialis anterior muscle of 2-month-old *DMSXL* and control mice (*n* ≤ 6). *P* values were calculated using the Student’s *t* test. *P < 0.05, **P < 0.01, ***P < 0.001.

We investigated whether these results could be translated to additional disease models and the clinical setting. Therefore, we first measured the expression levels of several of the aforementioned genes in PBMCs from a cohort of patients with DM1 established in Guipuzcoa (Basque Country, Spain) (18, 36) (Supplemental Table 4). DM1 samples displayed lower expression levels of *CDK2* and *HMG2A*, involved in the cell cycle; *AURKB*, *CDC45*, and *CENPA*, in cell division; and *BRCA1*, *RAD50*, and *RAD51*, in DDR when compared with a group of healthy individuals of similar age (Figure 3A). On the contrary, the levels of *KCND2* were elevated in blood samples from patients with DM1 (Figure 3B). We also compared the expression of those genes taking into account the sex and the absence or presence of cancer, which have been shown to be important features in patients with DM1 (36), obtaining a similar pattern of expression in the majority of cases except *HMG2A*, *AURKB*, and *RAD50* (Supplemental Figure 2).

We studied whether those pathways could be linked to muscle biology. For this, we measured the expression of the previously identified genes in myoblasts isolated from the same patients and also in the tibialis anterior muscle from *DMSXL* mouse model carrying > 1,000 CTG repeats (20). Of note, we detected the same expression pattern in both human myoblasts (Figure 3C) and tibialis anterior muscle from the *DMSXL* mouse model (Figure 3, D and E), in which genes associated with cell cycle, cell division, cell replication, and DDR were significantly decreased in DM1 samples. These data translate the results of the transcriptome analysis to patient blood samples and to muscle biology.

### Proliferation and DDR are impaired in DM1-derived fibroblasts.

**Figure 4:**
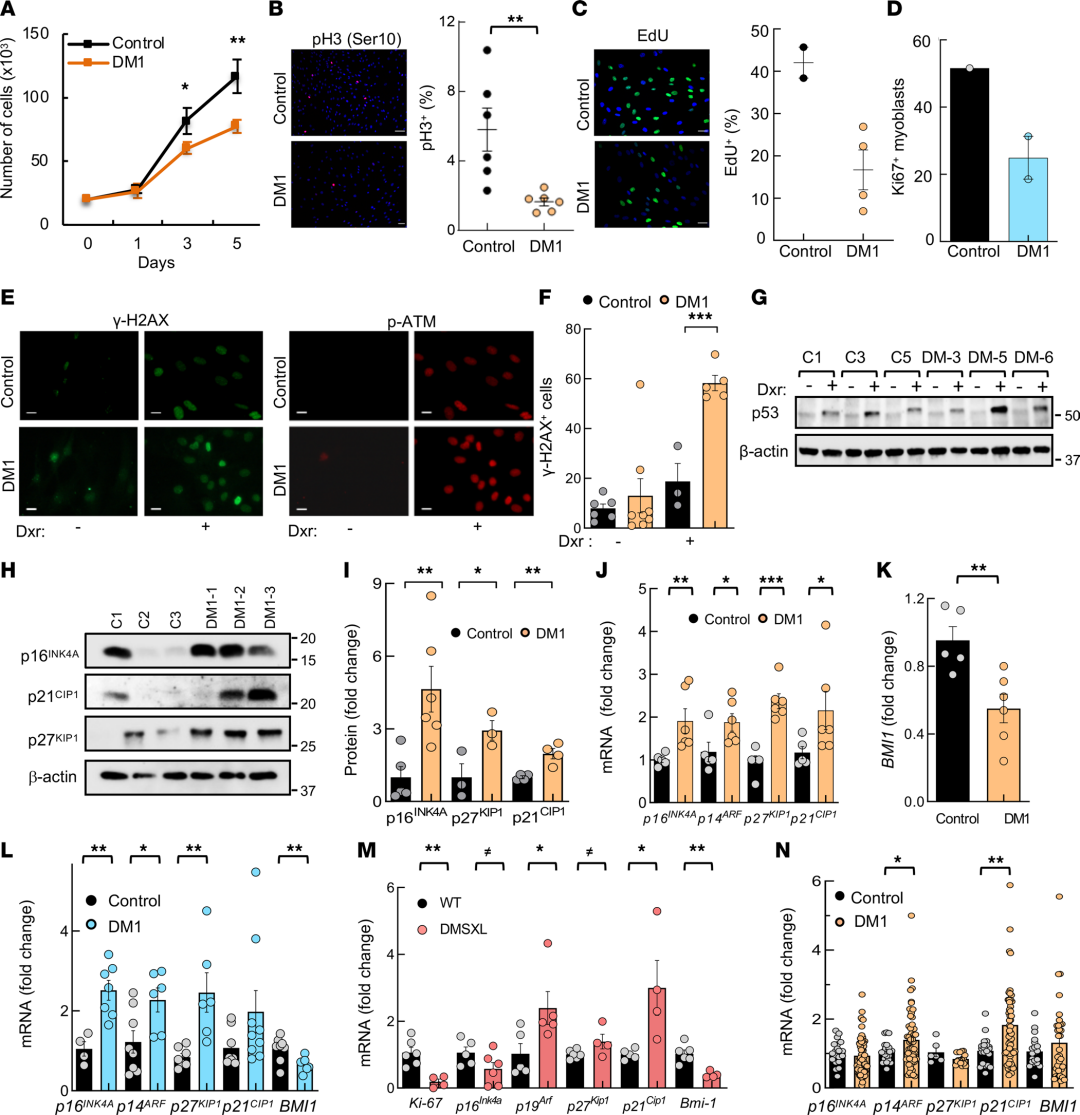
Proliferation and DNA damage response are impaired in DM1-derived fibroblasts. (**A**) Growth of DM1-derived fibroblasts (*n* = 6) compared with control fibroblasts (*n* = 5), analyzed as the number of cells at day 1, 3, and 5. (**B**) Representative image and quantification of pH3^+^ (Ser10^+^) cells in controls (*n* = 6) and DM1 fibroblasts (*n* = 6). Scale bar: 100 μm. (**C**) Representative image and quantification of EdU^+^ cells in controls (*n* = 2) and DM1 fibroblasts (*n* = 4). Scale bar: 50 μm. (**D**) Quantification of Ki67^+^ cells in controls and DM1 myoblasts (*n* = 2) at early passage (*n* ≤ 5). (**E**) Representative image and quantification of γ-H2AX^+^ cells and phospho-ATM detection in controls and DM1 fibroblasts in the absence or presence of doxorubicin (*n* ≥ 3). Scale bar: 50 μm. (**F**) Quantification of γ-H2AX^+^ cells in indicated conditions. (**G**) Representative immunoblot of p53 detection in controls and DM1 fibroblasts in the absence or presence of doxorubicin (*n* = 3). (**H** and **I**) Representative immunoblot and quantification of p16^INK4A^, p21^CIP1^, and p27^KIP1^ protein levels in DM1 compared with control fibroblasts (each point represents an independent sample). (**J**) mRNA levels of *p16^INK4A^*, *p14^ARF^*, *p27^KIP1^*, and *p21^CIP1^* in fibroblasts (*n* = 4 for controls and *n* = 6 for DM1). (**K**) mRNA levels of *BMI1* in the same fibroblasts as **J**. (**L**) mRNA levels of indicated genes in early passage myoblasts (*n* ≤ 7) derived from patients with DM1 and controls (each point represents an independent experiment). (**M**) Measurement of mRNA levels of indicated genes in tibialis anterior muscle of 2-month-old *DMSXL* and control mice (*n* ≤ 6). (**N**) *p16^INK4A^*, *p14^ARF^*, *p21^CIP1^*, *p27^KIP1^*, and *BMI1* mRNA levels in PBMCs from patients with DM1 (*n* ≥ 56) and controls (*n* ≥ 22). *P* values were calculated using the Student’s *t* test. ^≠^*P* < 0.1, *P < 0.05, **P < 0.01, ***P < 0.001.

The results presented above underscore the importance of cell proliferation and cell repair processes in DM1 pathology. Since previous studies have shown different results in regard to cell growth and cell proliferation in DM1 cells (17, 37, 38), we performed cell proliferation and DDR assays to functionally validate and extend our investigations. First, we found a significant decrease in the growth of DM1-derived fibroblasts, compared with control fibroblasts (Figure 4A). This was accompanied by a remarkable reduction in the number of cells positive for phospho-histone H3 (pH3) and EdU (Figure 4, B and C). In line with the lower proliferative capacity of DM1 fibroblasts, myoblasts also showed a lower number of Ki67^+^ cells (Figure 4D). Correlation analysis between pH3 numbers in DM1 fibroblasts and the number of CTG expansions and the Muscular Impairment Rating Scale (MIRS) revealed an inverse correlation between them, with strong coefficients (–0.657 and –0.525, respectively) but without reaching statistical significance (Supplemental Figure 3, A and B). In contrast, no clear association was detected with donor age (Supplemental Figure 3C).

To analyze cell repair and DDR processes, we determined the number of cells positive for γ-H2AX, a marker of the response to DNA damage (39). DM1 fibroblasts tended to have a higher number of γ-H2AX^+^ cells at an early passage (Figure 4, E and F). Treatment with doxorubicin, an inducer of DNA damage, increased γ-H2AX expression in both control and DM1 cells — but to significantly higher levels in the latter (Figure 4, E and F). In line with this, phospho-ATM — as well as p53 — levels were also elevated after doxorubicin treatment of DM1 fibroblasts (Figure 4, E–G, and Supplemental Figure 4A).

To further characterize cell cycle and cell proliferation, we measured p16^INK4A^, ARF (p14^ARF^ in humans and p19^ARF^ in mouse), and p21^CIP^, critical mediators of RB and p53 pathways (40), as well as p27^KIP1^. All 4 CDK inhibitors had elevated expression, at both the protein and mRNA levels in DM1 fibroblasts (Figure 4, H–J). Additionally, *BMI1*, a member of the Polycomb family that plays a role in cell proliferation and senescence through the repression of p16^INK4A^, ARF, and p21^CIP1^ (41, 42), showed significantly reduced expression in DM1 fibroblasts (Figure 4K). A similar pattern of expression of *CDK* inhibitors and *BMI1* was obtained in myoblasts (Figure 4L). We also detected elevated levels of *p19^Arf^*, *p21^Cip^*, and *p27^Kip^* and reduced levels of *Bmi1* and *Ki67* in muscle from *DMSXL* mouse model (Figure 4M), while *p14^ARF^* and *p21^CIP1^* presented elevated levels in DM1 PBMCs (Figure 4N). In contrast, *p16^INK4A^* and *BMI1* expression was altered depending on the sex and the presence of cancer in DM1 samples (Figure 4N and Supplemental Figure 4B); it was decreased and elevated, respectively, in female patients with increased cancer risk, but the opposite was observed in males, in agreement with a previous study (36). These data show that DM1 cells display cell cycle arrest and accumulate higher amounts of DNA damage after the induction of stress.

### DM1 fibroblasts undergo premature senescence.

**Figure 5:**
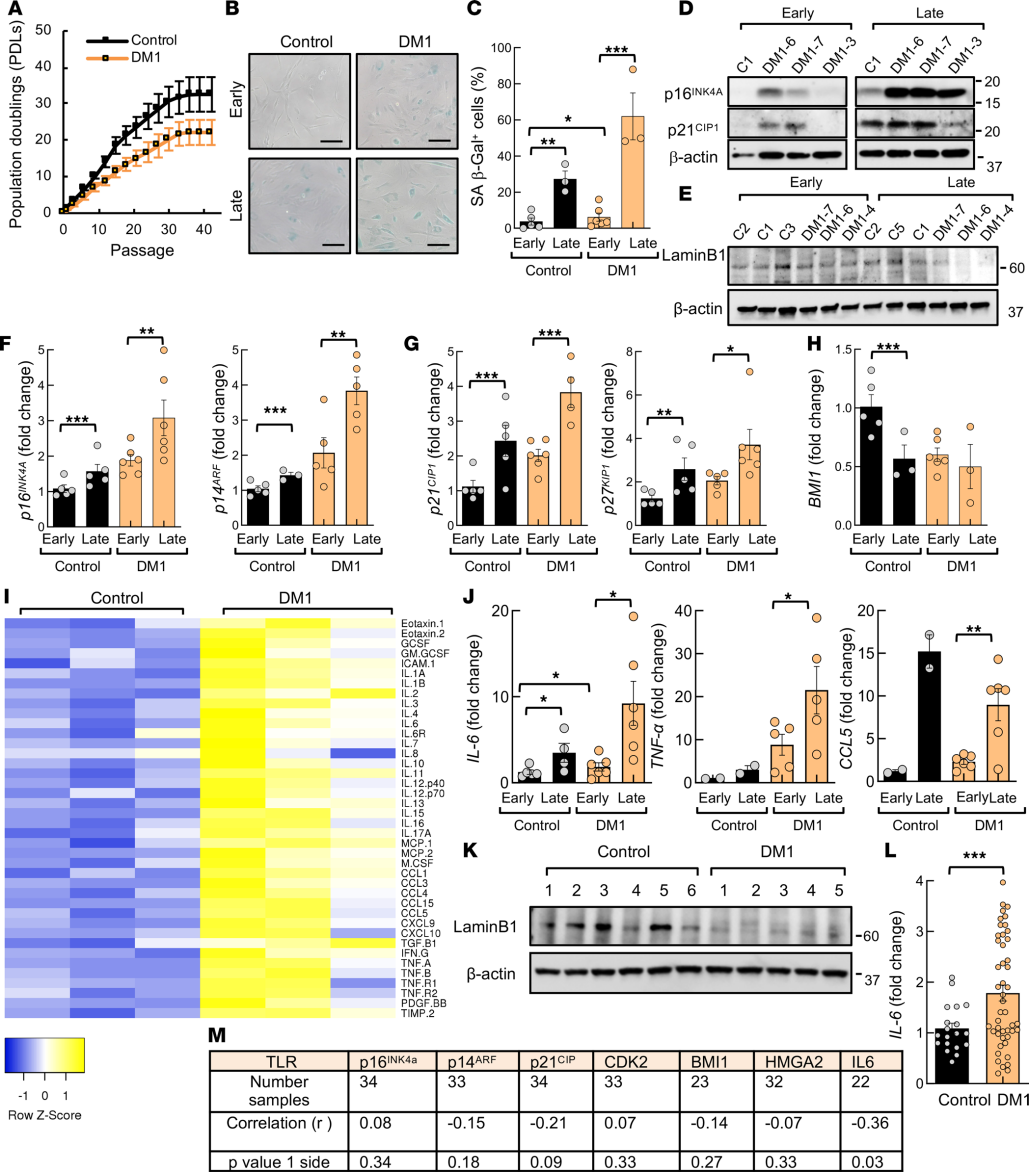
DM1 fibroblasts undergo premature senescence. (**A**) Results of 3T3 protocol where replicative senescence was induced in fibroblasts by seeding at a density of 30 × 10^3^ cells per 10 cm^2^ every 3 days. Population doubling (PDL) of DM1 cells (*n* ≥ 5) and controls (*n* = 4) was calculated at each passage. (**B** and **C**) Representative images and quantification of SA–β-galactosidase^+^ cells at early (between 3 and 10) and late passage (between 35 and 40) in control and DM1 fibroblasts (*n* ≥ 5). Scale bar: 100 μm. (**D** and **E**) Representative immunoblot of p16^INK4A^, p21^CIP1^, and Lamin B1 proteins at early (between 5 and 10) and late (between 35 and 40) passages in DM1 relative to control fibroblasts (*n* ≥ 4). (**F**–**H**) mRNA levels of *p16^INK4A^*, *p14^ARF^*, *p21^CIP1^*, *p27^KIP1^*, and *BMI1* in DM1 and controls (*n* ≥ 4), at early and late passages. (**I**) Antibody array of soluble factors secreted by control and DM1 fibroblasts. For each cell culture, control signals were averaged and used as the baseline. Signals above baseline are shown in yellow; signals below baseline are shown in blue. The heatmap indicates fold changes from baseline between DM1 (*n* = 3) and control fibroblasts (*n* = 3). (**J**) *IL6*, *TNFα*, and *CCL5* mRNA levels at early and late passages (*n* ≥ 4). (**K**) Representative immunoblot of Lamin B1 proteins in PBMCs from DM1 and controls (*n* ≥ 5). (**L**) Measurement of *IL6* mRNA in PBMCs derived from patients with DM1 (*n* ≥ 56) and controls (*n* ≥ 22). (**M**) Pearson’s correlation analysis between expression of senescence markers and leukocyte telomere length in patients with DM1. Telomere length was measured in DNA samples extracted with Qiagen in a previous study (18). *P* values were calculated using the Student’s *t* test with *P* value corrected for FDR. *P < 0.05, **P < 0.01, ***P < 0.001.

Cellular senescence is a state of stable and irreversible cell cycle arrest, in which cells remain metabolically active, but they can no longer replicate in response to different stresses (40, 43). Next, we investigated whether the accumulated deregulation of cell cycle, replication, division, and DDR could trigger the acquisition of a permanent senescent state in DM1. Thus, we performed a serial passage protocol and counted the number of population doublings (PDLs) after approximately 40 passages. DM1 fibroblasts displayed a decreased division rate, required a lower number of divisions and passages to undergo senescence, and stopped dividing faster compared with control cells (Figure 5A). This was accompanied by the presence of a higher number of senescence-associated β-galactosidase^+^ (SA–β-gal^+^) cells in DM1 in early passages but especially in late passage with the proportion of SA–β-gal^+^ cells reaching over 60% in DM1 (Figure 5, B and C). DM1 cells were also more enlarged, adopted a flattened morphology, and had a larger volume than control fibroblasts at advanced passage (Figure 5B). At the molecular level, the accumulation of components of the p16^INK4A^/Rb and ARF/p53/p21^CIP1^ pathways is required to promote temporary and permanent cell cycle arrest, stop cell division, and maintain a senescent state. Thus, we studied their expression and found that *p16^INK4A^*, *p14^ARF^*, *p21^CIP1^* levels, as well as *p27^KIP1^*, were increased at advanced passage, with higher levels in DM1 fibroblasts (Figure 5, D–G). On the contrary, the reduction of BMI1 and Lamin B1 are also biomarkers of senescence. In particular, loss of Lamin B1 has been postulated to be more specific biomarker than p16^Ink4a^ in DM1, as its downregulation was independent of the repeat length (17). The levels of Lamin B1 and *BMI1* were lower in DM1 fibroblasts in both early- and late-passage conditions (Figure 5, E and H).

Persistent DNA damage signaling triggers the secretion of several proinflammatory cytokines, a phenotype termed senescence-associated secretory phenotype (SASP), in which IL-6 plays a particularly relevant role (44, 45). To evaluate and compare the inflammatory state of control and DM1 fibroblasts, we performed a cytokine array analysis of the supernatants of both genotypes. We found a completely different pattern of the secretory phenotype between control and DM1 fibroblasts, with elevated expression of multiple proinflammatory cytokines (IL-6, IL-1α, TNF-α, IL-1β, IFN-γ), chemokines (CCL5), and other factors in DM1 cells at early passage (Figure 5H). We used qPCR to confirm the elevated expression of *IL6*, *TNFα*, and *CCL5* at the mRNA level in DM1 fibroblasts (Figure 5I). Further analysis of the cytokine and chemokine expression profile showed the elevation in *IL6*, *TNFα*, and *CCL5* mRNA levels in late passages, and it was particularly exacerbated in DM1 fibroblasts (Figure 5I), providing additional evidence of the accumulation of senescent cells and the acquisition of the SASP in DM1 fibroblasts.

In order to determine the translation of the senescent phenotype from in vitro to in vivo samples, we measured the expression of Lamin B1 and IL-6 in blood samples and found lower levels of Lamin B1 in PBMCs derived from patients with DM1 compared with those from healthy donors (Figure 5, J and K). On the contrary, *IL6* levels were elevated in DM1 samples (Figure 5L and Supplemental Figure 4C). Telomere shortening triggers the accumulation of DNA damage and senescence (43). We and others previously detected an accelerated attrition in telomere length in DM1 cells in vitro and in vivo, (14, 18). Taking advantage of this analysis, we performed a correlation analysis between telomere length and expression of *p16^INK4A^*, *p14^ARF^*, *p21^CIP^*, *CDK2*, *HMGA2*, and *IL6* in PBMC samples from patients with DM1 in vivo. Of note, there was an inverse correlation between telomere length and expression of *IL6* and possibly *p21^CIP^* (Figure 5M). Thus, DM1 PBMCs with shorter telomeres display higher levels of *IL6* and *p21^CIP^*. Taken together, our results illustrate the activation of senescence programs in DM1 cells in vitro and in vivo.

### BMI1 overexpression rescues impaired phenotypes of DM1 fibroblasts.

**Figure 6:**
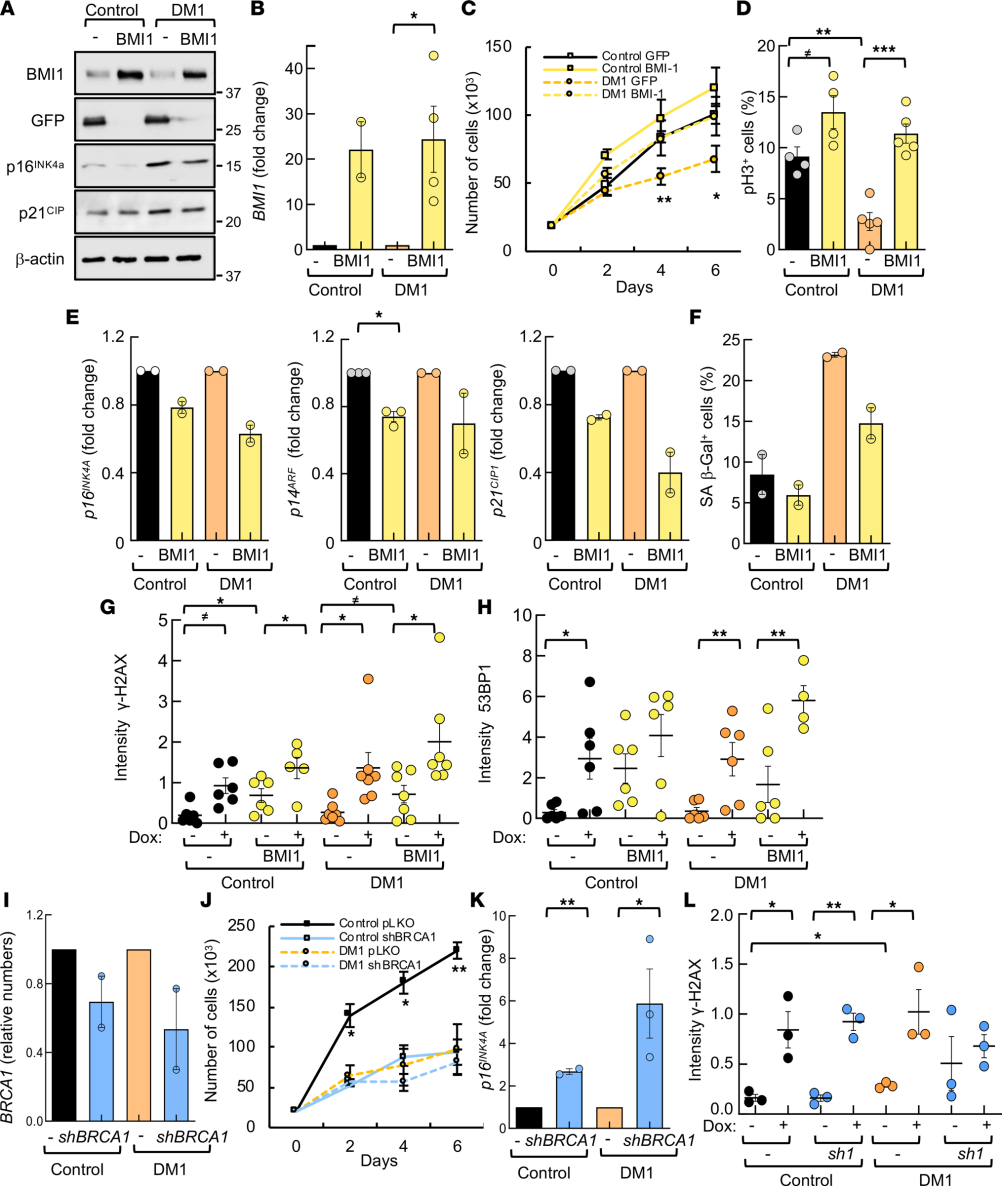
BMI1 pathway and DNA damage response regulate several phenotypes of DM1 fibroblasts. (**A**) Western blot of indicated proteins in control and DM1 fibroblasts infected with empty vector (–) or construct encoding *BMI1* (BMI1). Representative immunoblots of 3 independent experiments are shown. (**B**) *BMI1* mRNA levels in DM1 fibroblasts infected with empty vector or overexpressing *BMI1* compared with control fibroblasts (*n* = 2 experiments with 2 independent biological samples each). (**C**) Cell growth of different genotypes at the indicated time points (*n* ≥ 5). (**D**) Quantification of pH3^+^ cells in the indicated genotypes (*n* = 4). (**E**) mRNA levels of *p16^INK4A^*, *p14^ARF^,* and *p21^CIP1^* in DM1 (*n* = 3) and control fibroblasts (*n* = 2). (**F**) Quantification of SA–β-galactosidase^+^ cells in indicated genotypes. Results are average of 2 independent experiments with 2 independent samples each. (**G**) Quantification of γ-H2AX integrated fluorescence density at indicated genotypes in the absence or presence of doxorubicin (each point represents an independent sample; *n* ≥ 5). (**H**) Quantification of 53BP1 integrated fluorescence density in controls and DM1 fibroblasts in the absence or presence of doxorubicin (each point represents an independent sample; *n* ≥ 5). (**I**) *BRCA1* mRNA levels in DM1 and control fibroblasts infected with empty vector (*pLKO*, or *–*) or with a short hairpin (*shBRCA1*) (*n* = 2). Relative expression to *pLKO* condition in each genotype. (**J**) Cell growth of different genotypes at the indicated time points (*n* ≥ 2). (**K**) mRNA levels of *p16^INK4A^* in DM1 and control fibroblasts of the indicated genotypes (*n* ≥ 2). (**L**) Quantification of γ-H2AX integrated fluorescence density in controls and DM1 fibroblasts in the absence or presence of doxorubicin (*n* = 3). *P* values were calculated using the Student’s *t* test with *P* value corrected for FDR. ^≠^*P* < 0.1, *P < 0.05, **P < 0.01, ***P < 0.001.

Our previous results show that *BMI1* expression is decreased in DM1 cells in vitro. To determine whether BMI1 was involved in the reduced proliferative capacity of DM1 cells, we overexpressed it in control and DM1 fibroblasts, via lentiviral infection of a construct encoding the full length of the *BMI1* sequence. After confirming the increase in BMI1 expression at the protein (Figure 6A) and mRNA levels (Figure 6B), we performed functional studies and found that *BMI1* restoration rescued the impaired cell growth and proliferation of DM1 fibroblasts, to a similar extent to control cells (Figure 6, C and D, and Supplemental Figure 5A). In parallel, *BMI1* restoration reduced levels of p16^INK4A^, p14^ARF^, and p21^CIP1^ (Figure 6, A and E). Moreover, it also decreased the number of SA–β-gal^+^ cells (Figure 6F) and increased nuclear accumulation of γ-H2AX and 53BP1 proteins after treatment with doxorubicin (Figure 6, G and H, and Supplemental Figure 5, B and C). However, DMPK levels did not seem to be clearly altered with BMI1 overexpression (Supplemental Figure 5D). These results reveal that ectopic *BMI1* restored DM1-associated cellular and molecular alterations.

To determine whether impaired DNA repair machinery could be responsible of the phenotypes observed in DM1 cells, we knocked down BRCA1 expression with a short hairpin (*shBRCA1*) via lentiviral infections in control and DM1 fibroblasts. Silencing of *BRCA1* (Figure 6I) promoted a significant decrease in cell growth (Figure 6J) and an increase in *p16^INK4A^* and γ-H2AX levels in both control and DM1 fibroblasts (Figure 6, K and L, and Supplemental Figure 5E). Several genes involved in cell cycle, cell division, and DDR were also significantly altered in DM1 cells with *BRCA1* knockdown (Supplemental Figure 6, A–C). These results show that impaired DNA repair machinery is responsible, at least in part, for the phenotypes in DM1 cells.

### Senotherapy rescues DM1-associated impairments in vitro and in vivo.

**Figure 7:**
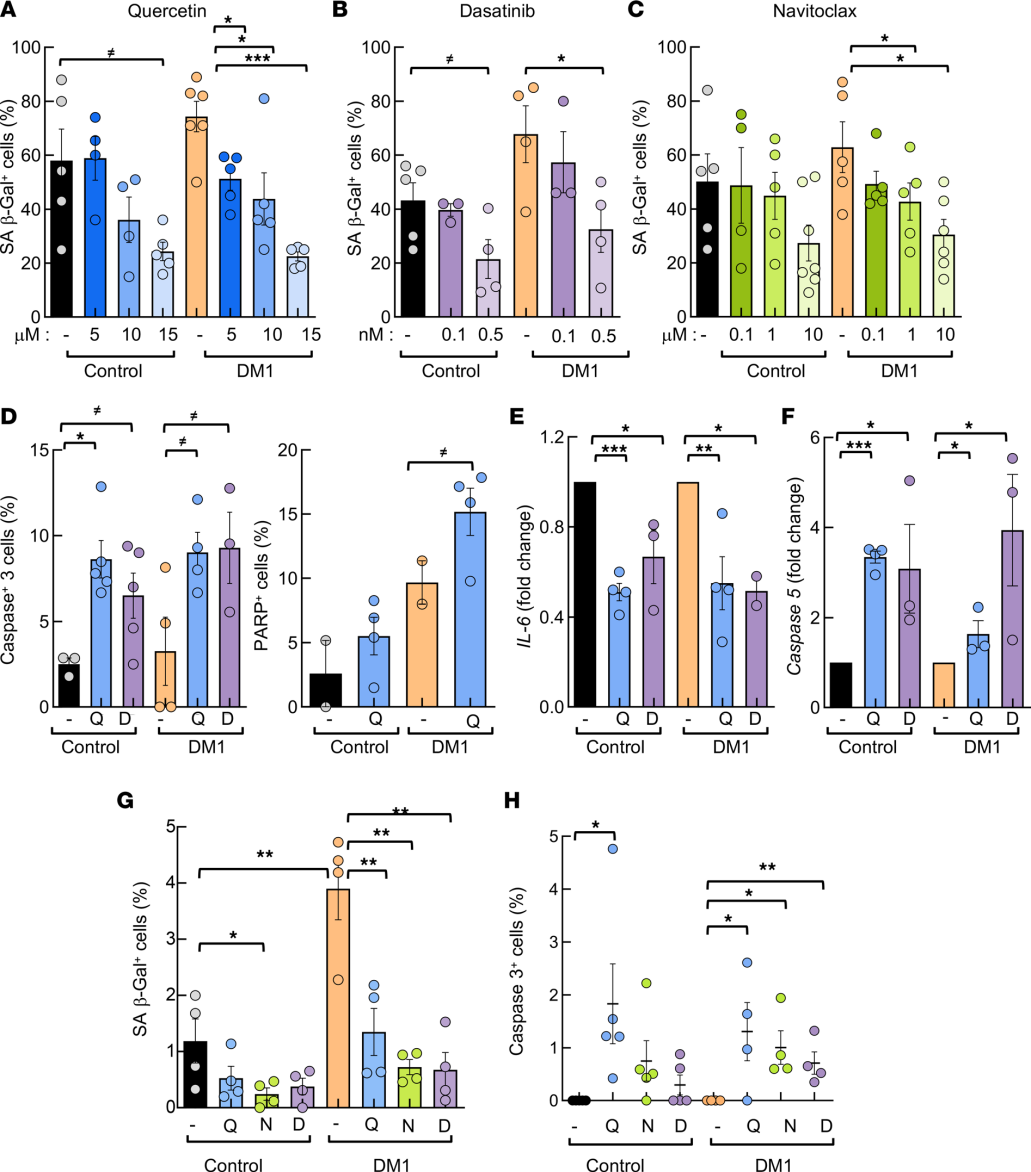
Senotherapy restores multiple deficiencies in DM1 fibroblasts in vitro. (**A–C**) Quantification of SA–β-galactosidase^+^ cells, in absolute numbers, in late-passage (*n* ≥ 40) DM1 and control fibroblasts treated for 72 hours (*n* ≥ 6) with 5, 10, or 15 μM Quercetin, where 15 μM Quercetin treatment decreased the number of positive cells from 58% to 36% in controls and from 74 to 22% in DM1 (**A**); 0.1 or 0.5 nM Dasatinib, where 0.5 nM Dasatinib decreased them from 43% to 26% in controls and from 67 to 32% in DM1 (**B**); or 0.1, 1, or 10 μM Navitoclax, where 10 μM Navitoclax decreased them from 50% to 27% in controls and from 62 to 26% in DM1 (**C**). (**D**) Quantification of apoptosis measured as active caspase-3 and cleaved-PARP at late passage (*n* ≥ 40) after treatment with Quercetin or Dasatinib for 3 days (*n* = 3). (**E** and **F**) mRNA levels of *IL6* and *caspase-5* in late-passage fibroblasts treated with Quercetin or Dasatinib for 3 days. Each point represents an independent experiment (*n* = 3). (**G**) Quantification of SA–β-galactosidase^+^ cells, in absolute numbers, in early-passage (*n* ≥ 5) DM1 and control fibroblasts treated for 72 hours (*n* ≥ 5) with 15 μM Quercetin, 0.5 nM Dasatinib, or 10 μM Navitoclax. (**H**) Quantification of active caspase-3^+^ cells in early passage (*n* = 1-5) DM1 and control fibroblasts treated for 72 hours (*n* ≥ 4) with 15 μM Quercetin, 0.5 nM Dasatinib, or 10μ M Navitoclax. *P* values were calculated using the Student’s *t* test with *P* value corrected for FDR. ^≠^*P* < 0.1, *P < 0.05, **P < 0.01, ***P < 0.001.

Senotherapy is a potentially novel therapeutic strategy that aims to permanently remove senescent cells, clear immune-mediated senescent cells, and neutralize the SASP (46). Next, we investigated the efficacy of this therapy against DM1. We treated late-passage DM1 and control fibroblasts with 3 independent senolytic compounds, Quercetin, Dasatinib, and Navitoclax, for 72 hours. First, we quantified SA–β-gal staining and observed a dose-dependent decrease in the number of positive cells in control and DM1 fibroblasts after treatment with each of the 3 senolytic compounds independently (Figure 7, A–C). In particular, 10 μM Quercetin or Navitoclax and 0.5 nM Dasatinib resulted in an over 50% reduction in SA–β-gal^+^ cells in DM1 and around 40% in controls (Figure 7, A–C, and Supplemental Figure 7A). We also found that the number of pH3^+^ cells decreased (Supplemental Figure 7B), whereas caspase-3 and PARP^+^ cells increased significantly in response to senolytic treatment (Figure 7D), thus confirming the death of senescent DM1 fibroblasts. These functional data were further validated at the molecular level, as *IL6* expression decreased to approximately 50%, whereas caspase-5 expression increased up to 4-fold after the treatment of DM1 and control fibroblasts with senolytics (Figure 7, E and F).

We performed similar experiments in early-passage fibroblasts. In this context, senolytic treatment also decreased the number of SA–β-gal^+^ cells (Figure 7G) and increased the number of caspase-3^+^ and PARP^+^ cells to a similar extent in control and DM1 cells (Figure 7H and Supplemental Figure 7C). In line with this, Quercetin also increased the number of apoptotic cells and decreased proliferation in myoblast cultures (Supplemental Figure 7, D–F). However, the effects were lower at early passage than at late passage. These results confirm that senolytics selectively killed senescent cells in control and, particularly, DM1 fibroblasts.

**Figure 8:**
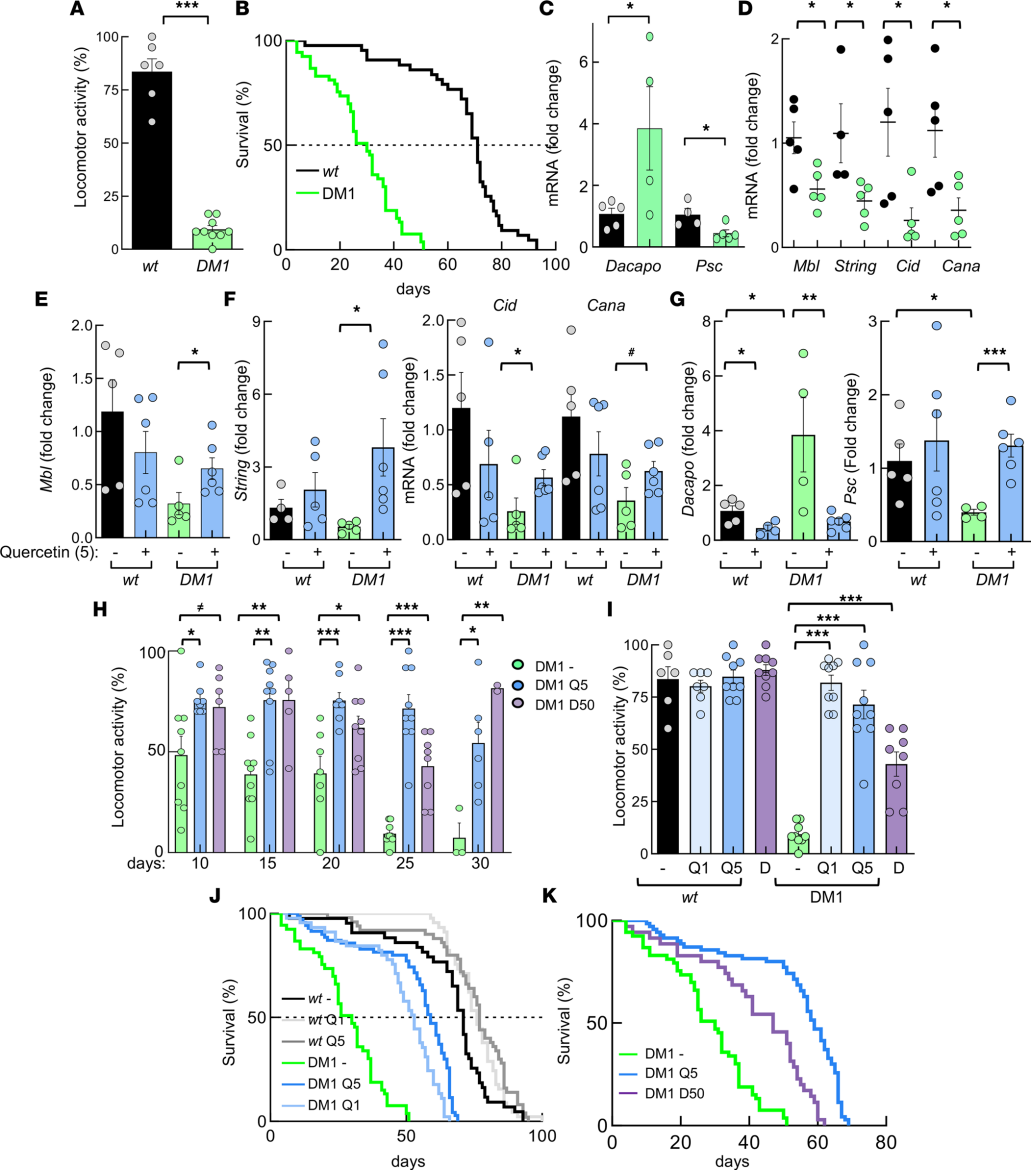
Senotherapy restores multiple deficiencies in DM1 fruit flies in vivo. (**A**) Locomotor activity obtained in *REC2 Drosophila* model of DM1 (*n* = 53) and controls (*n* = 46) at 25 days. (**B**) Survival curve of DM1 flies and controls. Median is 30 versus 71 days: *P* = 0.0001. (**C**) mRNA levels of *Dacapo* and *Psc* in thorax of DM1 and control fruit flies (*n* ≥ 4, each point represents a pool of 12 flies). (**D**) mRNA levels of indicated genes in DM1 and control fruit flies (*n* ≥ 4). (**E**–**G**) mRNA levels of indicated genes in DM1 and control fruit flies (*n* ≥ 4) in the absence or presence of 5 mM Quercetin. (**H**) Locomotor activity obtained in *REC2* *Drosophila* DM1 model nontreated (*n* = 55) or in presence of 5 mM Quercetin (*n* = 46) and 50 nM Dasatinib (*n* = 9) at the indicated time points. (**I**) Locomotor activity of nontreated DM1 (*n* = 9) and control (*n* = 6) flies or in the presence of 5 mM Quercetin (control, *n* = 7; DM1, *n* = 9) and 50 nM Dasatinib (control, *n* = 9; DM1, *n* = 8), respectively. (**J**) Survival curve of non-treated control (*n* = 45) and DM1 (*n* = 53) flies or in presence of 1 mM (DM1, *n* = 45; control, *n* = 50) and 5 mM Quercetin (DM1, *n* = 70; control, *n* = 45). *P* <0.0001 in the 2 doses of Quercetin in DM1 flies. Median in WT was 71, 77, and 76 days in nontreated, Q1, and Q5, respectively, and median in DM1 was 30, 59, and 53 in nontreated, Q1, and Q5, respectively. (**K**) Survival curve of DM1 flies nontreated (*n* = 53) or in presence of 5 mM Quercetin (*n* = 70) and 50 nM Dasatinib (*n* = 36). Median is 30 versus 59 versus 47 days and *P* < 0.0001 with both senolytics. *P* values were calculated using the Student’s *t* test with *P* value corrected for FDR, except for survival curves, which were completed with log-rank test. *P < 0.05, **P < 0.01, ***P < 0.001.

We aimed to validate the in vitro results in a *Drosophila melanogaster* model of DM1 that expresses 480 interrupted CTG repeats (21). First, we found that DM1 flies present impaired locomotor activity (Figure 8A) and reduced lifespan (Figure 8B) compared with control flies lacking CTG repeats, indicative of an accelerated aging phenotype. In line with this idea, the expression of the markers of senescence *Dacapo* (homolog of p21^CIP^/p27^KIP^) and *Psc* (homolog of BMI1) was elevated and reduced, respectively, in DM1 flies (Figure 8C). Moreover, DM1 flies showed reduced levels of *Mbl*, *String* (CDC45), *Cid* (CENPA), and *Cana* (CENPE) (Figure 8D), further validating the results obtained in the transcriptome study. In this context, 5 mM Quercetin rescued the expression of *Mbl*, *String*, *Cid*, and *Cana* (Figure 8, E and F). Similarly, the levels of *Dacapo* and *Psc* were restored after treatment with Quercetin (Figure 8G).

Finally, we measured locomotor activity and longevity in the absence or presence of 1 and 5 mM Quercetin and 50 nM Dasatinib. Both senolytics significantly increased the motility of DM1 flies at all studied times (Figure 8H). In particular, at 25 days, the percentage of flies moving was 9% in nontreated DM1 versus 71% with 5 mM Quercetin and 42% with 50 nM Dasatinib (Figure 8I). Even a low concentration of 1 mM of Quercetin promoted similar positive effect on locomotor activity of DM1 flies, whereas none of those concentrations altered the motility of WT flies (Figure 8I). Moreover, the senolytics extended the longevity of DM1 flies by increasing the median survival from 30 to 59 days with 5 mM Quercetin (log-rank test; *P* < 0.0001) and to 47 with 50 nM Dasatinib (*P* < 0.0001) (Figure 8, J and K). As with locomotor activity, senolytics did not significantly affect the survival of WT flies (Figure 8J and Supplemental Figure 8). These results confirm that senotherapy efficiently and selectively rescues a phenotypic spectrum of DM1 characteristics in patient-derived cells and in an in vivo model of the disease.

## Discussion

DM is a rare, clinically variable disease with no currently available treatment to slow or stop the progression of the disease. Supportive treatments, preventative measures, and clinical surveillance are the only options available for patients with DM1 (47). The variety of symptoms that develop patients with DM1 strongly resemble signs of accelerated aging (17, 23). Similarly, deregulation of several hallmarks of aging has been associated with the pathophysiology of DM1 in different human cell types (23). Transcriptomic analysis from cultures of primary fibroblasts derived from patients with DM1 identified dysregulation in pathways from various steps of cell cycle, cell division, cell replication, and cell repair, which are all relevant processes related to aging. The detailed impact of such deregulation on DM1 pathophysiology requires further investigation. However, preliminary evidence indicates that some of the processes, such as DNA repair, play a role in DM1 pathogenesis. In support of this, additional studies have shown that DNA repair affects repeat instability (48), and our functional studies revealed that DM1 fibroblasts show enhanced accumulation of DNA damage and that *BRCA1* mediates several cellular phenotypes. Previous studies detected normal cell growth in DM1 fibroblasts (41, 42) and myoblasts (49). In contrast, we observed that DM1 fibroblasts displayed reduced proliferation and cell growth. The age and health status of donors, the expansion of the triplet, the isolation of cells from DM1 or DM2 patients, culture conditions, the methodology, and experimental approach are characteristics linked to the proliferative capacity of fibroblasts; they might explain the differences, as they are different between both studies or have not been analyzed in all cases. Moreover, we also detected reduced proliferative capacity of myoblasts derived from the same individuals in vitro. In agreement with this, additional groups detected reduced proliferative capacity in DM1 cells of muscle origin in vitro (14, 15, 17, 50, 51). The decreased expression of critical genes of cell cycle, cell division, cell replication, and cell repair was also detected in myoblasts and PBMCs from patients, as well as in muscle from *DMSXL* mice and thorax of *REC2 Drosophila* model, rendering the results from sources such as in silico transcriptome analysis, cultured cells,muscle biology, and clinical samples.

Cellular senescence involves a series of progressive and phenotypically diverse cellular states acquired after an initial growth arrest (40, 52). Previous studies detected premature senescence in DM1 cells of muscle origin in vitro and linked it to *p16^INK4A^* (14, 17). In line with this, we also found the existence of senescence and a role of p16^INK4A^ pathway in vitro; however, our results show a more complex status, with some differences between in vitro and in vivo samples.

First, transcriptomic and molecular studies identified the impairment of cell cycle control, cell division, and DNA replication and repair pathways together with higher p16^INK4A^, ARF, p53, and p21^CIP1^ in early-passage DM1 fibroblasts, indicative of cell cycle arrest and the initiation of the senescence process. Validation of transcriptomic results in myoblast cultures and samples from animal DM1 models, as well as human PBMCs, provide evidence that the deregulation of those processes is a previous step for the onset of senescence. The exposition to culture stress promoted the continuous activation and exacerbated accumulation of p16^INK4A^/RB and ARF/p53/p21^CIP^ pathways at late passage. DM1 fibroblasts also accumulated additional markers of senescence, such as SA–β-gal, BMI1, and p27^KIP1^, although the latest also marks quiescence (53), reduction of Lamin B1, and higher levels of several cytokines, which indicated the presence of SASP. The latest both effects have been suggested to be required for senescence progression (52). These alterations suggest that DM1 fibroblasts progressed and reached the deep or late stage of senescence (40, 52) in vitro.

**Figure 9:**
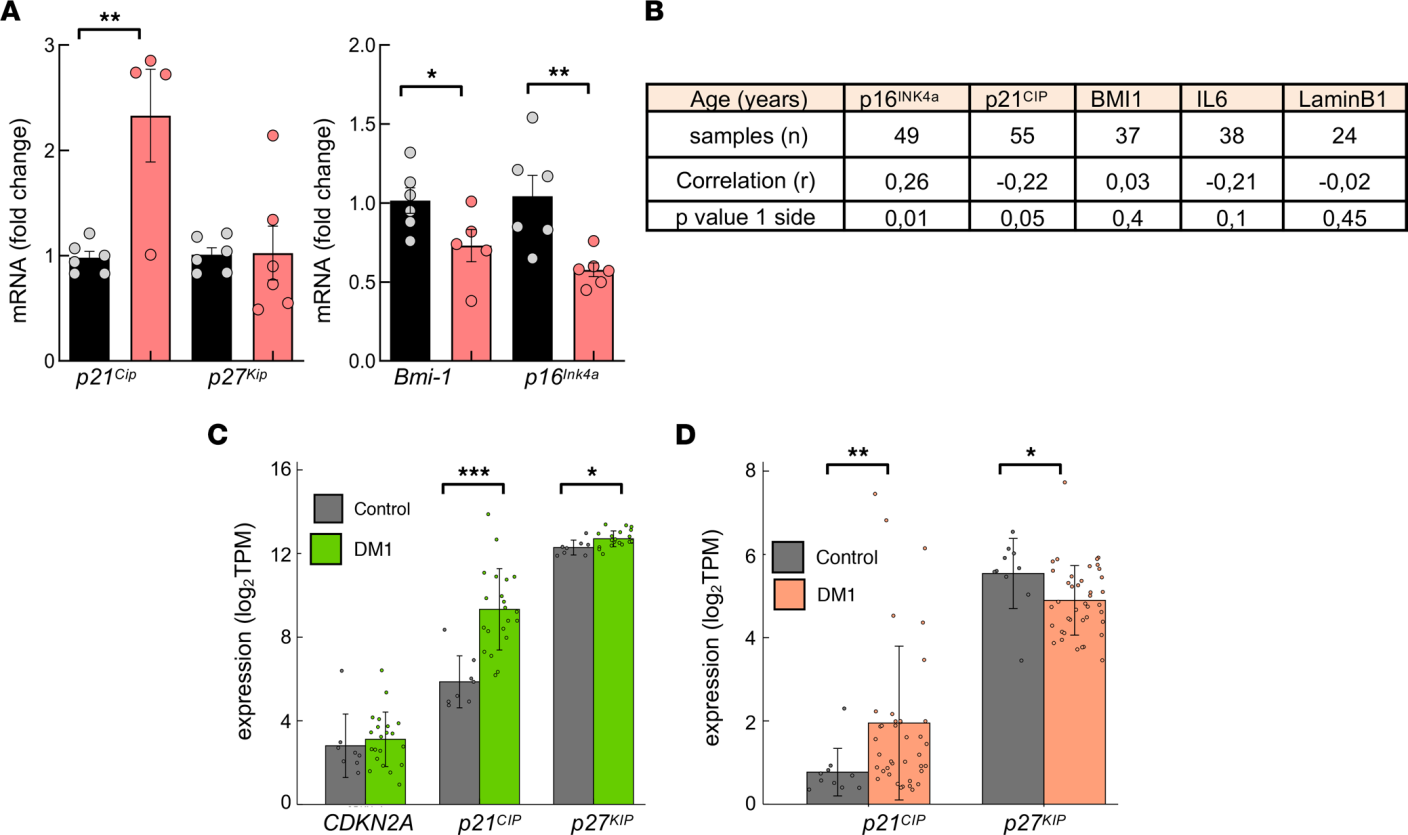
High levels of p21^CIP^ in DM1 samples in vivo. (**A**) Measurement of mRNA levels of indicated genes by qPCR in heart of 2-month-old *DMSXL* and control mice (*n* ≤ 6). Animals are the same as in Figure 4N. (**B**) Pearson’s correlation analysis between expression of senescence markers in PBMCs and age of patients with DM1. (**C**) Expression of indicated genes (in transcripts per million [TPM]) in cortex samples from patients with DM1 and downloaded from Gene Expression Omnibus (GEO; GSE157428). (**D**) Expression of indicated genes (in TPM) in cortex samples from patients with DM1 and downloaded from http://dmseq.org/data.html Statistical analysis was performed on log_2_-normalized expression, using 2-tailed Student’s *t* test for normally distributed gene expression data, tested with the Shapiro-Wilk test (*P* > 0.05). Otherwise, the Wilcoxon rank-sum test was used in genes (*CDKN2A* in **C** and *p21^CIP^* in **D**), where normality was rejected (*P* < 0.05).

In vivo, however, there is no homogeneous activation of cell cycle inhibitors with differences between sexes in the case of BMI1 and p16^INK4A^ expression in human PBMCs, no increase for p16^Ink4a^ and p27^KIP^ in different tissues of *DMSXL* mouse model (Figure 9A), and a lack of p16^Ink4a^ ortholog in *Drosophila*. The dynamic multistep theory of senescence proposes that the activation of p21^CIP^ is required for the onset and early stage of senescence, whereas p16^INK4A^ is associated with the deep or late stage of senescence, as it is activated under persistent stress and maintains a durable growth arrest (40, 52). Thus, the increased expression of *p21^CIP^*, but not *p16^INK4A^*, might suggest the accumulation of senescence in the early stage in DM1 cells in vivo. This is consistent with the age of patients (middle age) and *DMSXL* mice (2–3 months of age) included in the study. It is also supported by the elevated levels of *ARF* and *IL6* and the lower of Lamin B1. It is also in line with the inverse correlation between telomere length and the expression of *IL6* and *p21^CIP^*, but not *p16^Ink4a^*, as well as the positive and negative correlation of *p16^INK4A^* and *p21^CIP^* expression, respectively, with patient age (Figure 9B). The increased expression is also supported by results from muscle and brain samples from DM1 samples obtained from RNA-Seq data sets previously published from other research groups (54, 55), where p21^CIP^ expression is elevated but not CDKN2A or p27^KIP^ in DM1 samples (Figure 9, C and D). These results suggest that DM1 cells display enhanced activation and accumulation of senescence programs, but they are triggered gradually depending on the context and stress conditions. An additional study observed that senescent muscle cells from rats in vitro and in vivo share alternative splicing patterns with the *HSA^LR^* DM1 mouse model (56).

Senolytics are agents that induce apoptosis of senescent cells. The US Food and Drug Administration has already approved the treatment of Quercetin and Dasatinib, and preliminary results have shown that both are well tolerated and are effective in elderly individuals. Thus, the combination of these senolytics alleviated physical dysfunction in patients with idiopathic pulmonary fibrosis (57) and decreased the senescent cell burden in individuals with chronic kidney disease (58). Moreover, combined treatment with Quercetin and Dasatinib improved the health and lifespan of aged mice (59). Our results showed that Quercetin, Dasatinib individually, and Navitoclax (a) selectively eliminated senescent cells, (b) decreased the expression of SASP-related factors, and (c) rescued multiple phenotypes, including significant improvements in locomotor activity and longevity in the *REC2 Drosophila* model of DM1. Our study reveals that targeting senescent cells improves multiple cellular, molecular, and functional DM1 phenotypes, providing the proof of concept of the efficacy of senotherapy for the treatment of DM1 in the preclinical setting.

## Methods

### Samples from patients with DM1

#### Primary human fibroblast isolation and culture.

For the isolation of primary fibroblasts from healthy donors (age range, 27–52) and patients with DM1 (age range, 34–71), punch skin biopsies were cut into 2–3 mm^3^ fragments and placed on a surface moistened with modified Eagle’s basal medium, containing 13% newborn calf serum, 0.4% penicillin/streptomycin (Thermo Fisher Scientific), and 2 mM L-glutamine (Thermo Fisher Scientific). Flasks were incubated vertically for 3–6 hours at 37°C under 5% CO_2_ and then returned to the horizontal position. Human fibroblasts were cultured in DMEM (Thermo Fisher Scientific) containing 10% FBS (Sigma-Aldrich), 1% L-glutamine (Thermo Fisher Scientific), and 1% penicillin-streptomycin (Thermo Fisher Scientific). Fibroblasts were tested regularly for mycoplasma contamination and were negative. Seven cultures from different patients with DM1 and 6 from healthy controls were established and used for qPCR validation and functional studies (see Supplemental Table 1 for patient characteristics).

#### Primary human skeletal muscle isolation and culture.

Human proximal muscle biopsies were processed and cultured in monolayer. To obtain highly purified myoblasts, primary cultures were sorted by immunomagnetic selection based on the presence of the early cell surface marker CD56 (separator and reagents from Miltenyi Biotec). CD56^+^ cells were seeded at 2,500–3,000 cells/cm^2^ in culture medium for the myoblast stage, in cell culture plates previously treated with 0.5 % gelatin. Myoblast culture medium was composed of 65% DMEM and 21% M-199 (Sigma-Aldrich) and supplemented with 10% FBS, 1% insulin, 1% glutamine, 1% penicillin-streptomycin, 10 μg/μL EGF, and 25 μg/μL FGF. Muscle biopsy specimens were obtained from DM1 adult patients (donors of fibroblasts, as well as family members) and from healthy donors. These donors were healthy individuals who underwent surgery for bone fractures, and the muscle biopsies were obtained during this surgery. Samples were obtained from proximal limb muscles (biceps, deltoids, and triceps).

#### Blood samples and patient information.

Blood samples from healthy controls and patients with DM1 were obtained between 2012 and 2015 and were stored in the Basque Biobank (https://www.biobancovasco.org/en/). PBMCs isolation was performed between 2016 and 2017 using Lymphoprep (Axis-Shield). RNA was extracted using the miRNesay Mini Kit, followed by automated RNA extraction in the QIAcube (Qiagen), and protein extraction was performed following standard procedures. Data from patients with DM1 were retrospectively obtained from the medical records of the Guipúzcoa historical DM cohort, established in 1985 (36). From patients medical recorded, we selected sex, age at the time of the study enrollment, nucleotide expansion size (CTG triplets), disease severity assessed using the MIRS scale (60) at the time of the last visit, and cancer diagnosis. Supplemental Table 4 shows a summary of donor subject and patient characteristics. To measure the CTG expansion size in fibroblasts, we performed small-pool PCR, described elsewhere (61). Some modifications were applied: (a) fragments were amplified with Long Amp Taq (New England Biolabs) supplemented with 2% DMSO and 0,1% Tween20, and (b) DNA fragments were resolved by electrophoresis on a 1% agarose gel, followed by Southern blot. The mode allele was measured for each cell type by comparison against the molecular weight ladder, using GelAnalyzer 19.1 software.

### Animal models

Transgenic *Drosophila melanogaster* Mhc-Gal4 UAS-i(CTG)480 flies (21) were a gift from Rubén Artero (Valencia University, Valencia, Spain). Flies without any marker or balancer were selected for experiments to ensure the expression of the nucleotide repetition. Control flies were obtained crossing UAS-+-+ flies (a gift from Angel Cedazo-Minguez, Karolinska Institute, Stockholm, Sweden) and Mhc-Gal4 flies (purchased from Bloomington Stock Center, Indiana University, Bloomington, USA) to obtain UAS-+;MhcGal4 flies. Flies were housed at 23°C, 70% humidity, and 12-hour/12-hour light/darkness cycle. All crosses were carried out at these standard conditions with standard fly food or plus 1 or 5 mM Quercetin and 50 nM Dasatinib for treated groups. For the longevity assay, around 50 flies (5 females/tube) were selected from each group of study. Dead flies were counted every 2 days. The Kaplan-Meier method was used to plot the results. A log-rank test was used to analyze results, and Bonferroni correction was used for multiple comparisons. For locomotor activity, 5-, 10-, 15-, 20-, 25-, and 30-day-old flies were analyzed in groups of 5. They were placed in a tube with an 8 cm mark from the bottom. Assays were recorded and archived. The number of flies that passed the 8 cm mark in 10 seconds was counted (3 times/tube). mRNA was isolated from thorax of 12 flies per condition.

*DMSXL* mice were previously described (20). Tibialis anterior muscle from 6 *DMSXL* and 6 control 2-month-old littermates were a gift from Geneviéve Gourdon (INSERM, Paris, France).

### Transcriptomic study and data analysis

Microarrays were performed with RNA extracted from the fibroblasts from 6 patients with DM1 (3 male and 3 female; age 34–59 years) and 3 controls (2 male and 1 female; age 46–52 years) at early passage using TRIzol (Invitrogen). Large-scale gene expression analysis was performed using the Human Clariom D microarray (Affymetrix). RNA integrity value above 9 was confirmed using an RNA 6000 Nano kit (Agilent Technologies). A total of 300 ng of RNA from each sample was used for microarray analysis, following the manufacturer’s instructions.

#### Microarray data analysis.

Array normalization was performed using the Affymetrix Robust Multi-array Analysis (RMA) background correction model. To find the statistically significant differentially expressed genes (DEGs) between 2 groups of samples, we calculated the mean values of each probe across all the samples of each of the 2 groups. Next, we filtered out all the probes whose absolute value of difference of mean values between the 2 groups was less than a selection threshold θ_DEG_ = 2 (that corresponds to a fold change of 1 in a log_2_ scale), and we applied the Student’s *t* test. The multitest effect influence was tackled through control of the FDR using the Benjamini-Hochberg method for correcting the initial *P* values with significance threshold α_DEG_ = 0.01. The GO terms were taken from the curated collection of molecular signatures (gene set collection C5) of the Molecular Signatures Database (MSigDB; version 3.0). The significance of DEGs was analyzed using an enrichment approach based on the hypergeometric distribution to estimate the significance (*P* value) of the gene set enrichment. Data postprocessing and graphics were performed with in-house developed functions in Matlab (MathWorks).

### Data availability statement

The data discussed in this publication have been deposited in NCBI’s Gene Expression Omnibus (GEO) and are accessible through GEO series accession no. GSE142542.

### FISH

In total, 5 × 10^3^ fibroblasts were plated in 8-chamber slides. After an overnight (o/n) incubation, fibroblasts were fixed in 4% formaldehyde (Sigma-Aldrich) for 15 minutes, washed, and permeabilized by treatment with 70% ethanol o/n at 4°C. Cells were rehydrated for 5 minutes at room temperature, in 2× SSC (Sigma-Aldrich) with 50% formamide (Sigma-Aldrich); they were then incubated for 5 minutes at 80°C prior to hybridization. Then, cells were hybridized in an atmosphere saturated with 100% humidity o/n at 37°C. Hybridization buffer was composed of 10% dextran sulfate (Sigma-Aldrich), 2 mM vanadyl-ribonucleoside complex (Sigma-Aldrich), 0.02% RNase-free BSA (Sigma-Aldrich), 2× SSC, 50% formamide, and 0.1 μM Alexa Fluor 488 (CAG)x10 probe (Thermo Fisher Scientific). Then, fibroblasts were washed twice for 30 minutes with 2× SSC, 50% formamide at 37°C; stained with DAPI (1:600); mounted using Fluoro-Gel (17985-10, Aname); and examined using Zeiss LSM 900 confocal microscope. Images were processed using Zen Blue and ImageJ software (NIH).

### Proliferation and senescence studies

For cell growth assays, 3 × 10^4^ early-passage fibroblasts were seeded in 6-well plates in complete medium and counted at days 1, 3, and 5 using a light microscope.

To assess senescence, a serial passage protocol was performed. Human fibroblasts were cultured and passaged every 3 days. PDLs were calculated using the formula PDL = log_10_ (no. of cells at day 3/no. of cells at day 0)/log_10_. Additionally, senescence was studied at early (5–10 cell passages), intermediate (10–25 cell passages), and late passages (35–40 cell passages) by measuring SA–β-gal activity using the SA–β-gal Staining Kit (Cell Signaling Technology), according to the manufacturer’s protocol. At least 3 controls and DM1 samples were cultured simultaneously to complete these experiments. Fibroblast morphology was determined using an inverted microscope (Nikon Eclipse TS100) coupled with a digital camera (Nikon D90). Five photographs were taken in each culture, and their morphology was evaluated.

### Treatment with senolytics

Quercetin (Sigma-Aldrich), Navitoclax (Selleckchem), and Dasatinib (Sigma-Aldrich) were used to treat fibroblasts. For SA–β-gal measurement, 0.5 × 10^4^ cells were seeded in immunofluorescence chambers and then treated with 5, 10, or 15 μM Quercetin; 0.1, 1, or 10 μM Navitoclax; or 0.1 or 0.5 nM Dasatinib for 72 hours. For mRNA expression analysis, 10 × 10^4^ fibroblasts were seeded in p100 plates and then incubated with 15 μM Quercetin, 10 μM Navitoclax, or 0.5 nM Dasatinib for 48 hours. In these experiments, early passage was considered between 1 and 5, whereas late passage was over 40.

### mRNA expression analysis

Total RNA was extracted using TRIzol (Life Technologies). Reverse transcription was performed using random priming and the Maxima First Strand cDNA Synthesis Kit for qPCR, with dsDNase (Thermo Fisher Scientific), according to the manufacturer’s guidelines. qPCR was performed using Power SYBR Green PCR Master Mix (Thermo Fisher Scientific), 10 mM of each primer and 20 ng of cDNA, in a CFX384 thermocycler (Bio-Rad). Variations in RNA input were corrected by analyzing the expression of *GAPDH* (human), β-actin (mice), or rp49 (*Drosophila*) as a housekeeping gene. The ΔΔCt method was used for relative quantification. Primers sequences are presented in Supplemental Table 5.

Splicing assays were performed by reverse-transcription PCR (RT-PCR), using BIOTAQ DNA Polymerase (Bioline). The primers used have been described previously and were selected to give a length difference of 10%–25% between exon-inclusion and exon-exclusion products (62). PCR amplification was performed for 20–24 cycles, and PCR products were resolved on agarose gels, stained with GelRed Nucleic Acid Gel Stain (Biotium), visualized using an iBright FL1000 system (Invitrogen), and quantified using ImageJ (NIH).

### Western blotting and immunofluorescence analysis

Immunoblotting and immunofluorescence assays were performed following standard procedures. The following primary antibodies were used: anti-DMPK (1:200 and 1:500, sc-134319; Santa Cruz Biotechnology; 1:25, MANDM4 and MANDM13, DSHB), anti-MBNL1 (1:500, ab45899; Abcam), anti-CUGBP1 (1:500, ab9549; Abcam), anti–pH3 Ser10 (1:2000, ab14955; Abcam), anti-p27^KIP1^ (1:500, BD610241; BD Biosciences), anti-p16^INK4A^ (1:250, ab108349; Abcam), anti-p21^CIP1^ (1:500, sc-397; Santa Cruz Biotechnology), anti-BMI1 (1:500, 05-637; MilliporeSigma), anti-p53 (1:250, 2524, Cell Signaling Technology), anti–Lamin B1 (1:50, 12586, Cell Signaling Technology), anti-GFP (1:1,000, ab-6673; Abcam), anti–γ-H2AX Ser139 (1:250, 05-636; MilliporeSigma), anti-53BP1 (1:250, NB100-304; Novus Biologicals), anti–phospho-ATM Ser1981 (1:250, 13050, Cell Signaling Technology), anti–cleaved PARP1 (1:700, ab32064; Abcam), anti–active caspase-3 (1:500, AF835; R&D Systems), and anti–β-actin (1:10,000, AC-15; Sigma-Aldrich). For Western blotting, HRP-linked secondary antibodies were used (Cell Signaling Technology), and detection was performed by chemiluminescence using the Novex ECL Chemiluminescent Substrate Reagent Kit (Thermo Fisher Scientific) in an iBright FL1000 system (Invitrogen). For EdU assay, Click-iT EdU Cell Proliferation Kit with Alexa Fluor 488 dye was used (C10337, Invitrogen) following manufacture′s protocol. For immunofluorescence assays, nuclear DNA was stained with Hoechst 33342 (Sigma-Aldrich), and slides were examined using Zeiss LSM 900 confocal microscope or Nikon Eclipse 80i microscope.

To measure DNA damage and the DDR, fibroblasts were treated overnight with 1 μM doxorubicin (Sigma-Aldrich). For the fluorescence intensity measurement of γ-H2AX, 53BP1, DMPK, MBNL1, and CUGBP1, at least 100 cells were measured and an outline of the same size around the nucleus was used as the background fluorescence. Using ImageJ, we calculated the integrated intensity subtracting the background fluorescence for each fibroblast. For γ-H2AX, 53BP1, and CUGBP1 we measured nuclear fluorescence, and for DMPK and MBNL1, we measured nuclear and cytoplasmic fluorescence.

### Lentiviral transductions

For stable overexpression of BMI1, lentiviral transduction of primary fibroblasts was performed using a pCCL-BMI1 construct (a gift from Jacqueline Lees’s group, Institute of Integrative Cancer Research, Massachusetts Institute of Technology, Cambridge, Massachusetts, USA) and pCCL-GFP as a control. After incubation with polybrene (Sigma-Aldrich), cells were infected at a multiplicity of infection of 10 for 6 hours, and then medium without serum was replaced with normal medium. For *BRCA1* silencing, lentiviral transduction was performed using pLKO-shBRCA1#1 (this construct was a gift from Eros Lazzerini Denchi, Addgene, #44594) and the corresponding pLKO.1 puro (gift from Bob Weinberg, Addgene, #8453).

### Cytokine antibody arrays

Fibroblast cultures were washed and incubated in serum-free DMEM for 48 hours to generate conditioned medium (CM), which was then collected. The cells were then counted. CM was frozen at −80°C and analyzed using the Human Inflammation Array C3 (RayBiotech), following the manufacturer’s instructions. Briefly, CM was thawed and centrifuged for 5 minutes at 13,835.25*g* at room temperature. Array membranes were preincubated with 2 mL of blocking solution, incubated with CM overnight at 4°C, washed 5 times, and incubated with a biotin-conjugated antibody cocktail overnight at 4°C. After 5 washes, membranes were incubated with HRP-streptavidin for 2 hours at room temperature and then visualized using chemiluminescence detection in an iBright FL1000 system (Invitrogen). Signals were analyzed and normalized using ImageJ.

### Statistics

Data are presented as mean values ± SEM, with the number of experiments (*n*) in parentheses. Unless otherwise indicated, statistical significance (*P* values) was calculated using the 2-tailed Student’s *t* test with *P* value corrected for FDR. *P* < 0.05 was considered significant. Statistics are not be applied to experiments with *n* = 2. In the transcriptomics, gene set enrichment was calculated using the hypergeometric distribution. The multitest effect influence was corrected trough controlling the FDR using the Benjamini-Hochberg correction at a significance level α = 0.05. Correlation analyses were done using Pearson’s coefficient when samples were normally distributed or Spearman’s coefficient when samples were not normally distributed using GraphPad. Log-rank test was used for longevity studies.

### Study approval

This study was approved by the Donostia University Hospital Ethical Board (approval no. 15-57) and was conducted in accordance with the Declaration of Helsinki’s ethical standards. All subjects gave written informed consent before sample donation.

## Author contributions

MGP, ASA, and AAL performed all the experiments in fibroblasts. GG performed experiments in fruit flies. AS and MGP performed experiments in myoblasts. MGP and ASA are co–first authors; MGP is listed first due to starting the project, analyzing the results, and completing most of the figures. RFT and MZ collected clinical data and samples from the patients, evaluated the patients, and did the clinical experiments. JE performed bioinformatic analysis of public available datasets. GNG performed analysis of CTG expansion in fibroblasts. SMG performed telomere length measurement and correlation analysis with senescence markers. MJAB performed the transcriptomic analysis. AM and ALDM directed the project, contributed to data analysis, and wrote the manuscript.

## Supplementary Material

Supplemental data

## Version 1 08/30/2022

In-Press Preview

## Version 2 10/10/2022

Electronic publication
