# Supplementary material for: Senescence plays a role in myotonic dystrophy type 1
Source: JCI Insight. 2022 Oct 10;7(19):e159357. doi: 10.1172/jci.insight.159357 (PMC9675450; doi:10.1172/jci.insight.159357)
Supplement: Supplemental data [file jciinsight-7-159357-s109.pdf]

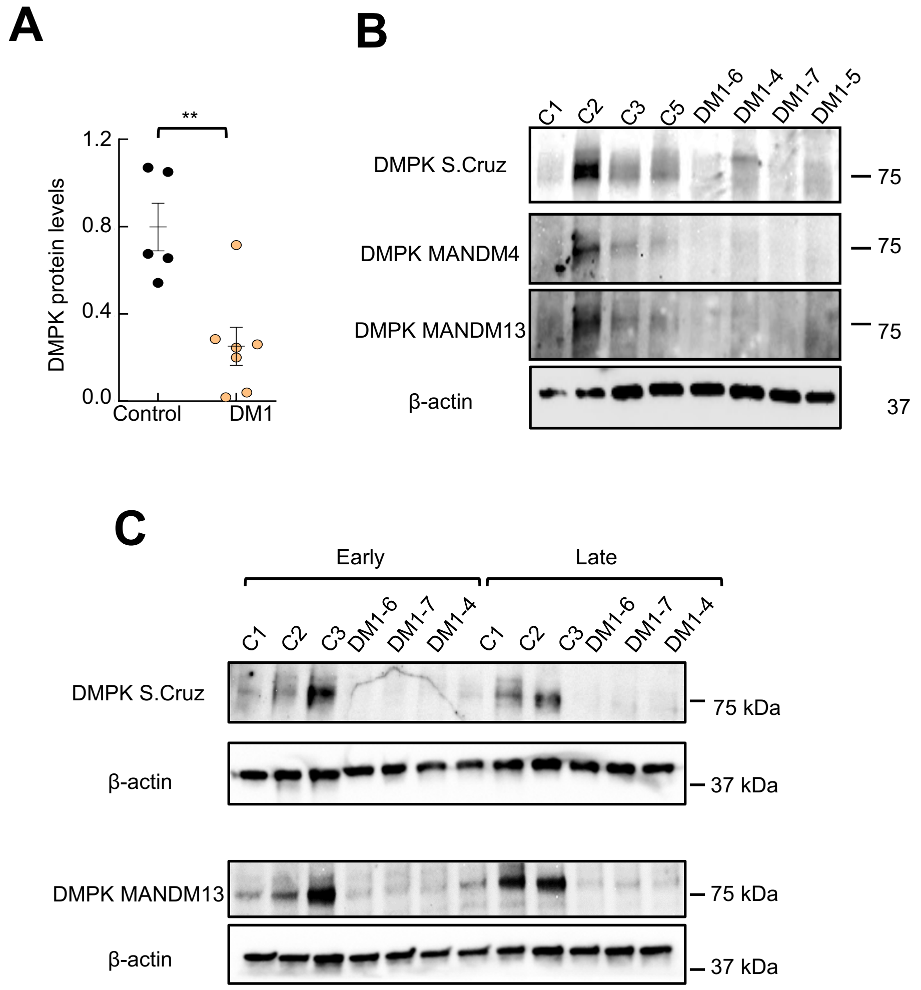

Figure 1. Expression of DMPK in fibroblasts derived from DM1 individuals.
A) Quantification of DMPK protein levels, stained with Santa Cruz Antibody, in fibroblasts derived from DM1 patients and controls (each point represents one independent sample. **B)** Representative Immunoblot of DMPK protein levels in fibroblasts derived from DM1 patients and controls stained with the indicated antibodies **C)** Representative Immunoblot of DMPK protein levels in fibroblasts derived from DM1 patients and controls at early (between 0-10) and late (between 35-40) passage stained with the indicated antibodies.

Fig. Supplementary 1 Garcia-Puga

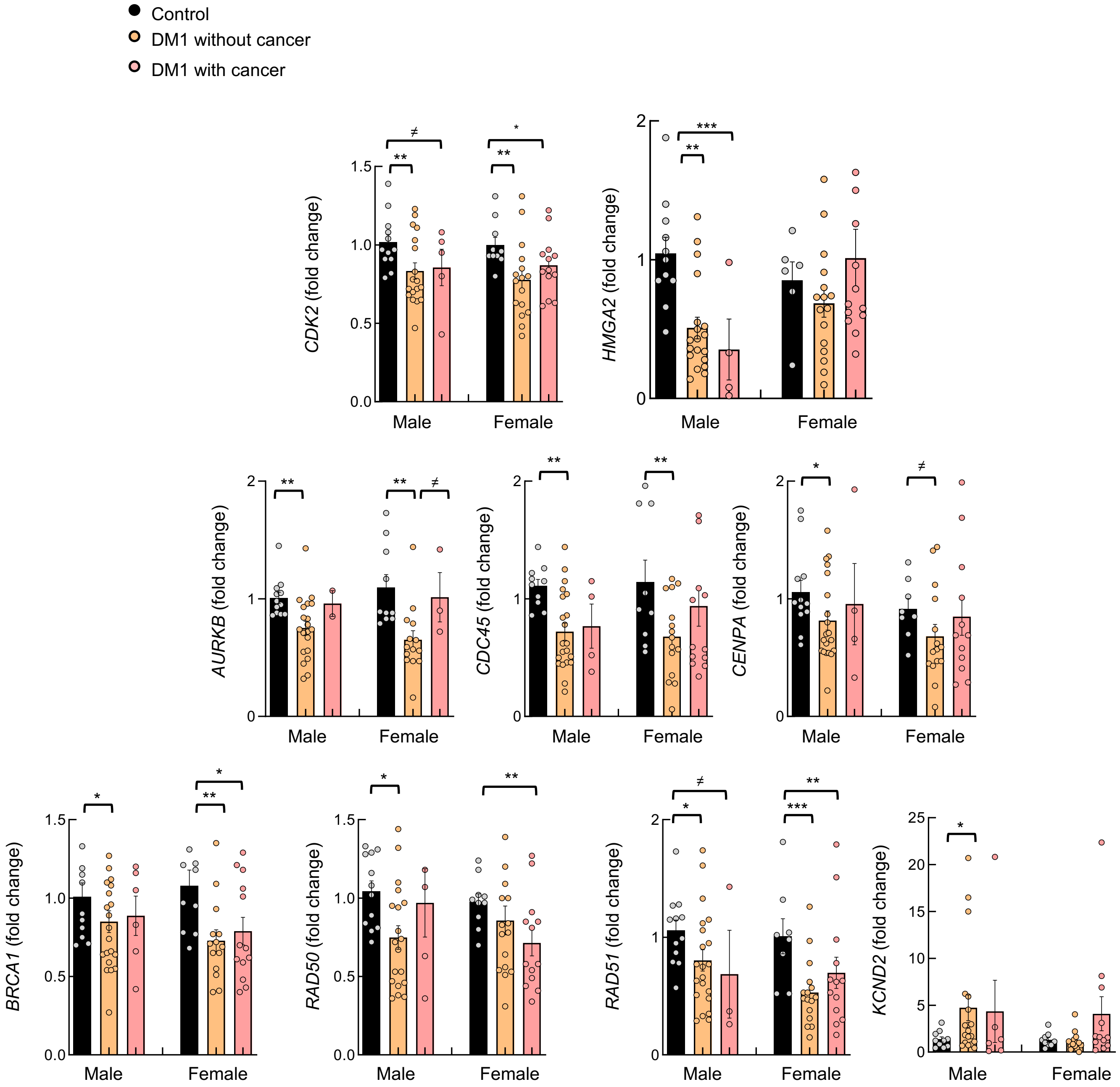

Figure 2. Genes involved in cell cycle, cell division, cell replication and DDR are diminished in PBMCs of DM1 individuals.
A) Measurement of mRNA levels of indicated genes by qPCR in PBMCs derived from DM1 patients (n≥56) and controls (n≥22). Both groups were divided by gender (DM1 male (n=27) and female (n=29) and controls male (n=12) and female (n=10) and, in the case of DM1, by the development of cancer (red; male (n=6) and female (n=13)) or not (orange; male (n=21) and female (n=16)).

Fig. Supplementary 2 Garcia-Puga

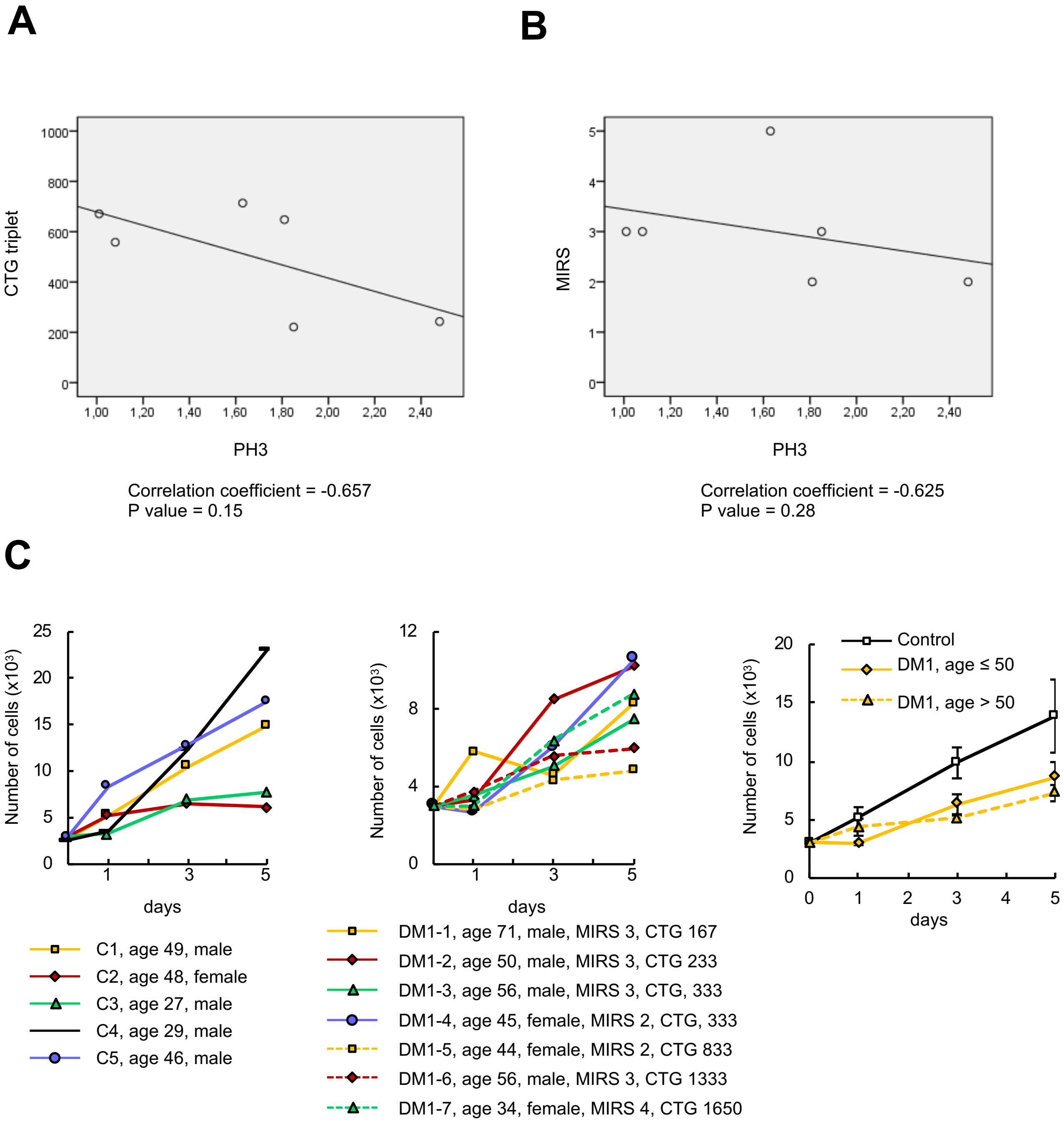

Figure 3. Correlation of proliferation with clinical characteristics.
A) Correlation analysis of phospho-Histone H3 numbers in DM1 derived fibroblasts and CTG triplet expansion. **B)** Correlation analysis of phospho-Histone H3 numbers in DM1 derived fibroblasts and MIRS scale. **C)** Cellular growth in control and DM1 fibroblasts stratified by donor age. Proliferation does not seem to be associated to age of individuals.

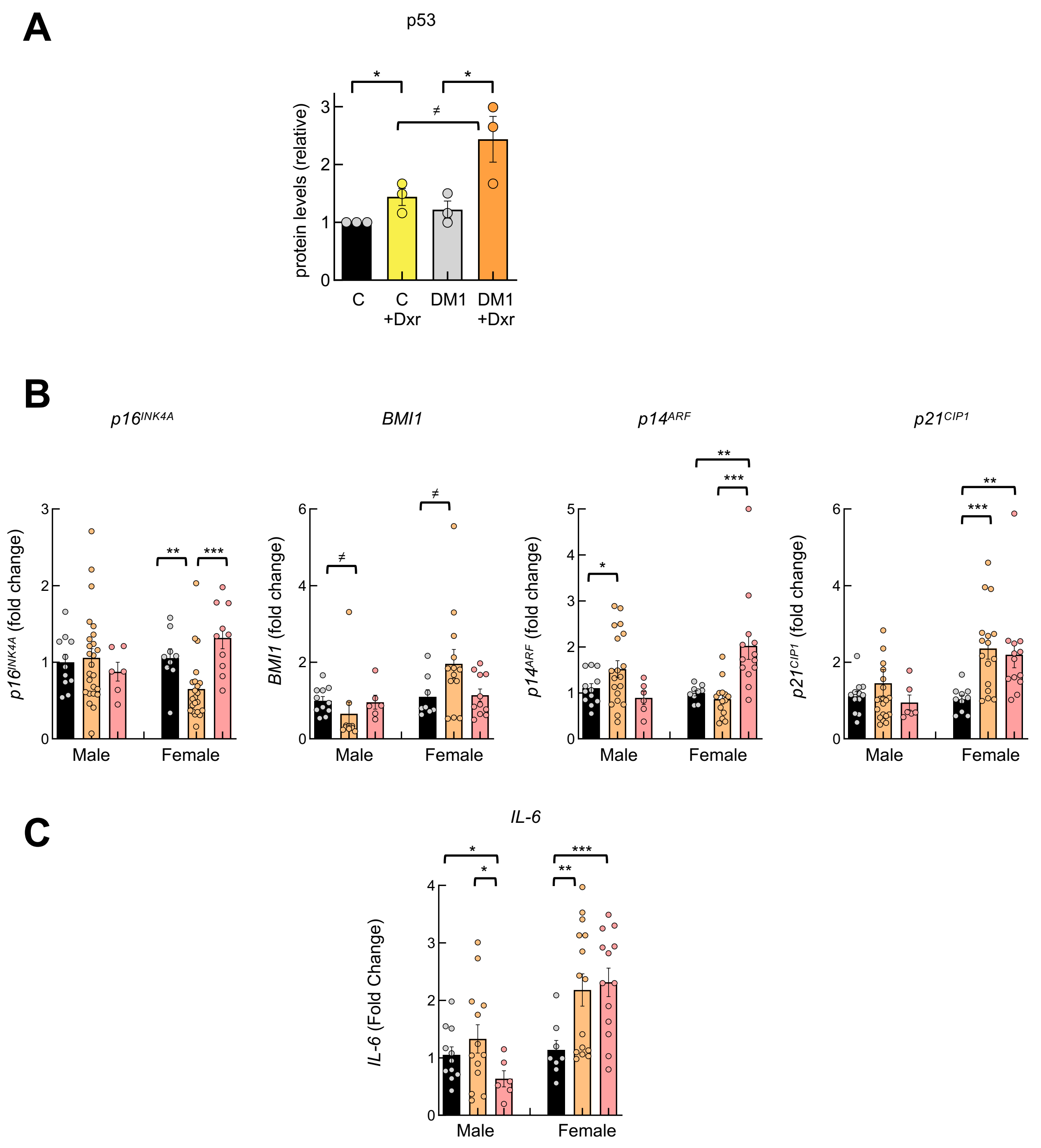

Figure 4. Expression of senescence markers p53, CDKIs and IL6 is altered in samples of DM1 individuals.
A) Quantification of p53 expression in controls and DM1 fibroblasts in the absence or presence of doxorubicin (n=3). Difference ≠ is =0.07. **B,C)** Measurement of mRNA levels of indicated genes by qPCR in PBMCs derived from DM1 patients (n≥56) and controls (n≥22). Both groups were divided by gender (DM1 male (n=27) and female (n=29) and controls male (n=12) and female (n=10) and, in the case of DM1, by the development of cancer (red; male (n=6) and female (n=13)) or not (orange; male (n=21) and female (n=16)).

Fig. Supplementary 4 Garcia-Puga

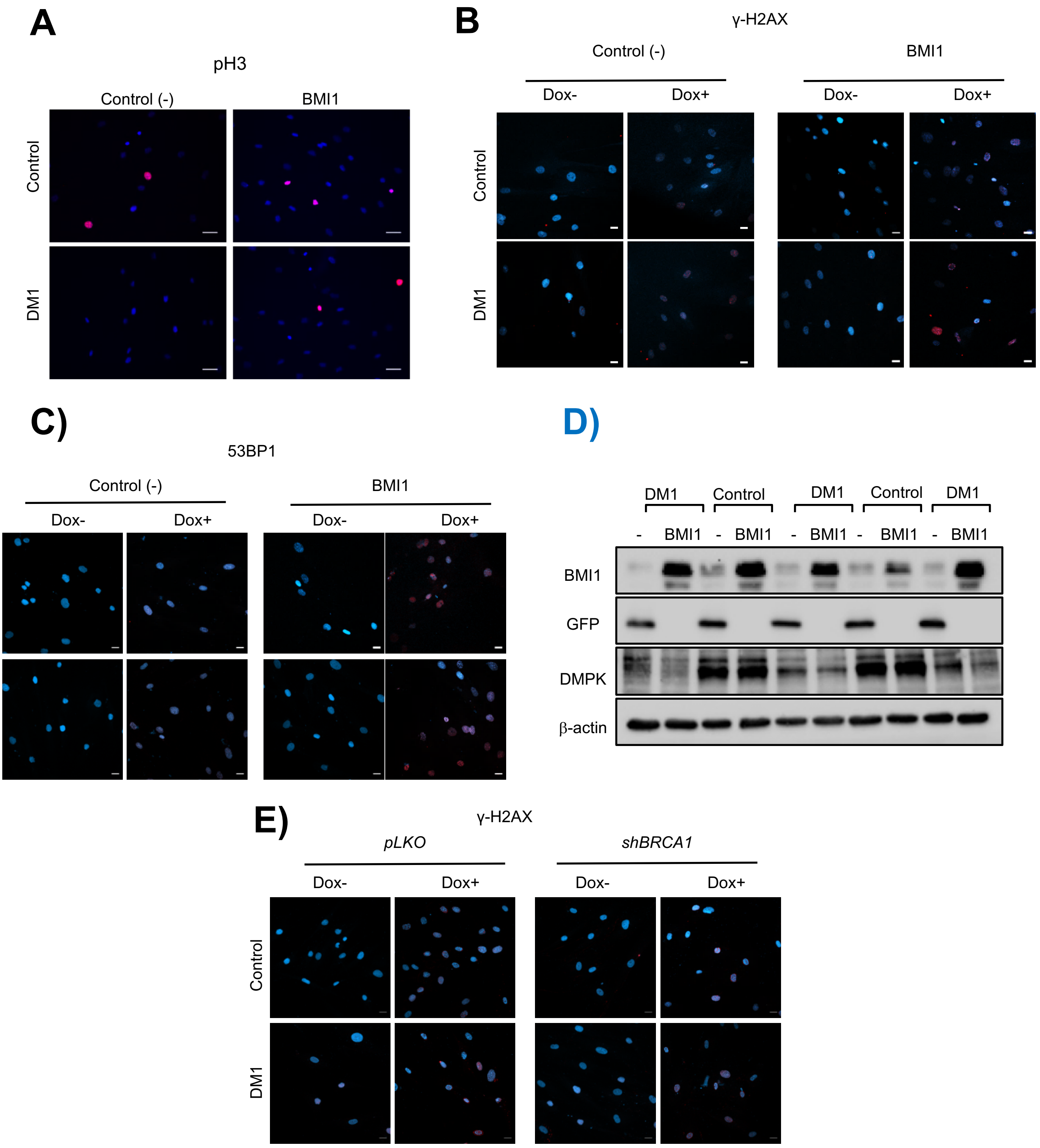

Figure 5. BMI1 and DDR play a role in DM1 cellular phenotypes.
A) Representative image of phospho-Histone H3 (pH3) positive cells in controls and DM1 fibroblasts infected with empty vector with GFP (-) or a plasmid encoding *BMI1* (BMI1). **B)** Representative image of γ-H2AX in controls and DM1 fibroblasts infected with empty vector (-) or *BMI1* in the absence (Dox-) or presence of doxorubicin (Dox+). **C)** Representative image of 53BP1 detection in controls and DM1 fibroblasts infected with empty vector (-) or a plasmid encoding BMI1 in the absence (Dox-) or presence of doxorubicin (Dox+). **D)** Western Blot of indicated proteins in control and DM1 cells infected with empty vector (-) or BMI1. **E)** Representative image of γ-H2AX-positive cells in controls and DM1 fibroblasts infected with empty vector (*pLKO*) or a short hairpin of BRCA1 (*shBRCA1*) in the in the absence (Dox-) or presence of doxorubicin (Dox+). Scale bar: 50 μm

Fig. Supplementary 5 Garcia-Puga

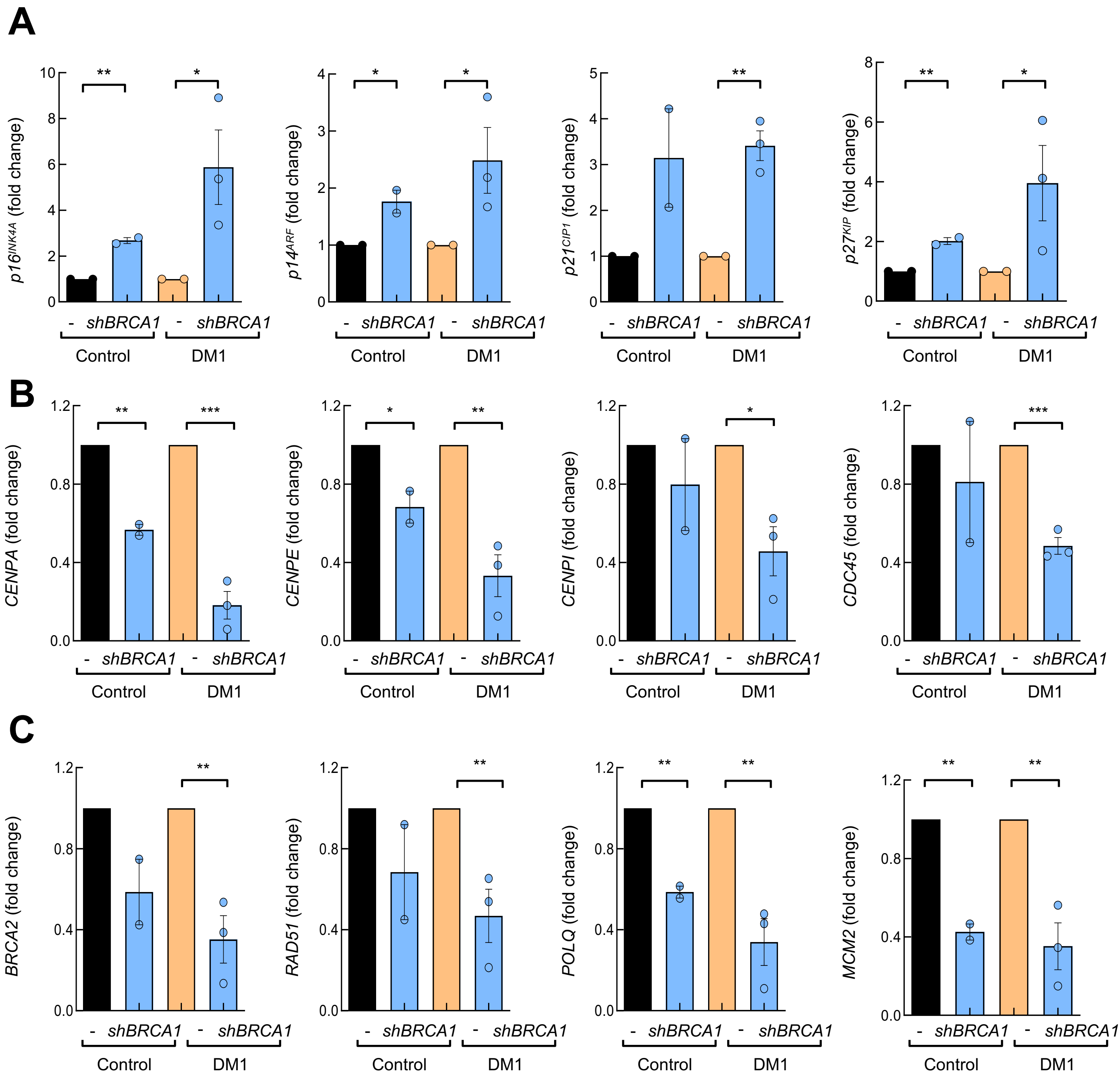

Figure 6. BRCA1 regulates the expression of relevant genes involved in cell cycle, cell division and DDR.
A-C) mRNA levels of genes involved in cell cycle, cell division and replication and DDR in DM1 and control fibroblasts of the indicated genotypes (n≥2).

Fig. Supplementary 6 Garcia-Puga

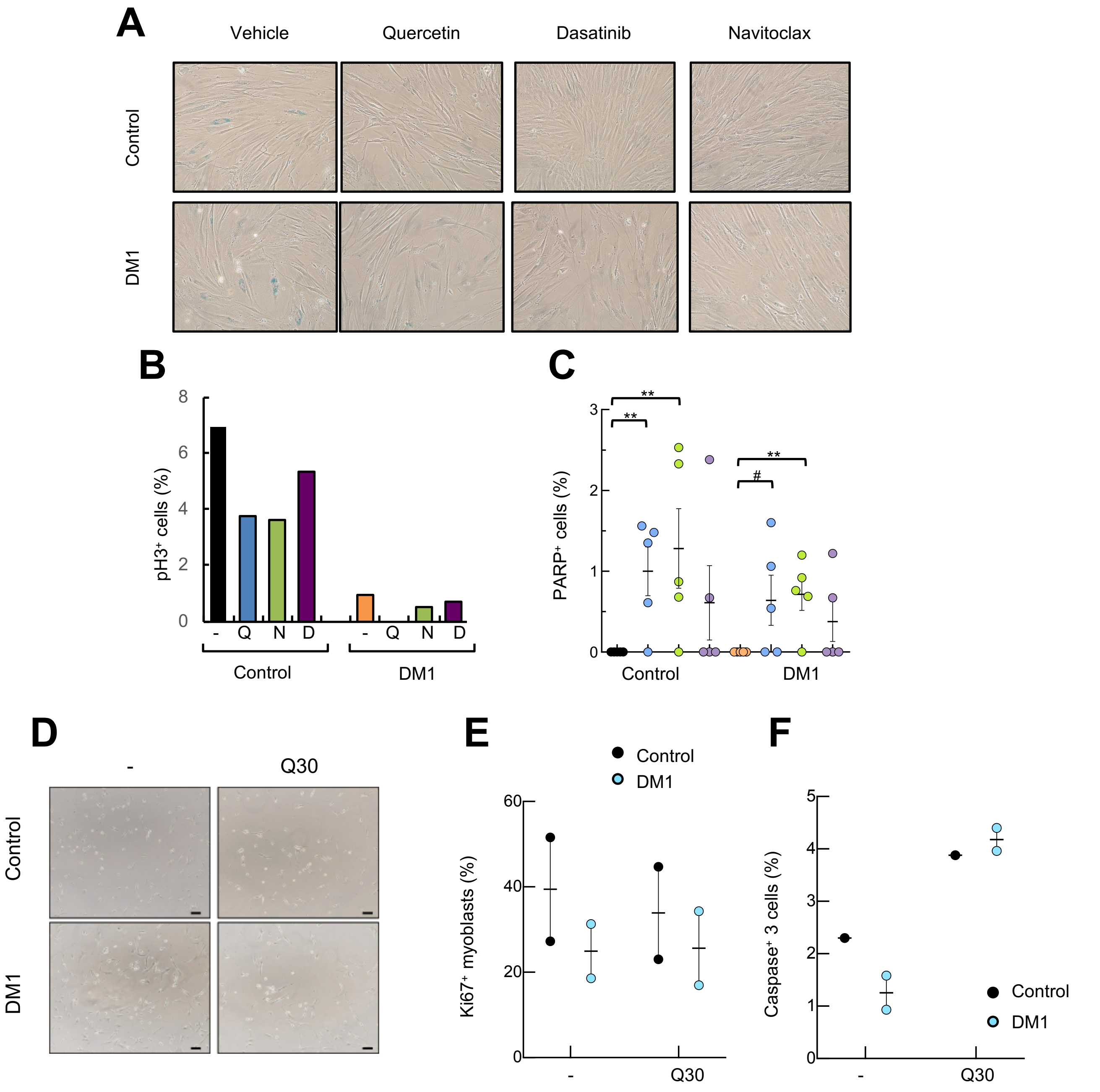

Figure 7. Senotherapy kills senescent cells in DM1 cells.
Representative images of SA β-galactosidase in control and DM1 fibroblasts at late passage in the absence or treated with 15 mM Quercetin, 0.5 nM Dasatinib or 10 mM Navitoclax for 3 days. **B)** Quantification of pH3 positive cells in fibroblasts in the absence or treated with 15 mM Quercetin, 0.5 nM Dasatinib or 10 mM Navitoclax at late passage. **C)** Quantification of PARP positive cells in fibroblasts at early passage in the absence or treated with 15 mM Quercetin, 0.5 nM Dasatinib or 10 mM Navitoclax. **D)** Representative images of SA β-galactosidase in myoblasts at early passage (3-5) in the absence or treated with 30 mM Quercetin. DM1 myoblasts present few SA β-galactosidase, which are not detected with Quercetin treatment. scale bar is 100 μm. **E)** Quantification of Ki67 positive cells in myoblasts at early passage (3-5) in the absence or treated with 30 mM Quercetin for 3 days. **F)** Quantification of Caspase 3 positive cells in myoblasts at early passage in the absence or treated with 30 mM Quercetin for 3 days.

Fig. Supplementary 7 Garcia-Puga

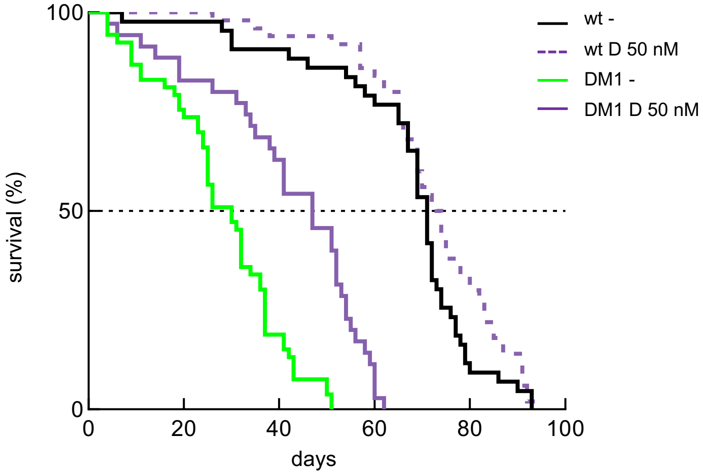

Figure 8. Dasatinib significantly extends longevity in *REC2 Drosophila* DM1 model *in vivo*.
Survival curve of non-treated control (n=45) and DM1 flies (n=53) or in presence of 50nM Dasatinib (Control, n=48 and DM1, n=36). Median survival in days is 73 *vs* non-defined *in controls (non-treated vs Dasatinib, p>0.05)* and 30 vs 47 in DM1 flies *(non-treated vs Dasatinib,,* p<0.0001)

Fig. Supplementary 8 Garcia-Puga

Supplemental table 1. Clinical characteristics of the individuals from whom fibroblasts were isolated and used in this study. Myoblasts were isolated from patient DM1-2 and DM1-7

Fibroblast Cell Line	Status	Gender	MIRS	CTG (n) in blood	Age at diagnosis (years)	CTG (n) in fibroblast (early passage)	CTG (n) in fibroblast (late passage)	Age at biopsy (years)
C1	Control	M						49
C2	Control	F						48
C3	Control	M						27
C4	Control	M						29
C5	Control	M						46
C6	Control	?						52
DM1-1	DM1	M	3	167	53	221	304	71
DM1-2	DM1	M	5	233	20	714	463	50
DM1-3	DM1	M	3	333	33	671	1287	56
DM1-4	DM1	F	2	333	41	243	1425	45
DM1-5	DM1	F	2	833	27	648	802	44
DM1-6	DM1	M	3	1333	20	588	1378	56
DM1-7	DM1	F	4	1650	12	750	866	34

MIRS (Muscle impairment rating scale)

Supplemental table 2. Gene Set Enrichment analysis (GSEA) and genes downregulated in DM1 derived fibroblasts. *p* –value shows significance of the gene set enrichment in DM1 compared to control fibroblasts.

Count	p-val	GSEA	Genes
**44/1037**	5.935e−08	CELL-CYCLE-PROCESS	AURKB BCL2L1 BRCA1 BRCA2 CDC45 CDCA3 CDK2 CDK6 2 DIAPH2 DTL EZR FBXO5 FGF10 FOXM1 GAS2L3 GINS1 GTSE1 HMGA2 KIFC1 MAP2K6 MCM3 MCM4 MCM8 MELK MKI67 NCAPG NCAPG2 NCAPH NEK2 NUF2 PIM2 PRIM1 PRKAR2B PRKCA RPA4 SGOL2 SKA1 SMC1A SPAG5 STMN1
**15/186**	5.930e−07	CONDENSED-CHROMOSOME	AURKB BRCA1 CDK2 CENPE CENPM CENPW MKI67 NCAPG NCAPH NEK2 NUF2 SGOL2 SKA1 SMC1A SPAG5
**33/749**	9.992e−07	MITOTIC-CELL-CYCLE	AURKB BCL2L1 CDC45 CDCA3 CDK2 CDK6 CENPE CENPW FBXO5 FGF10 FOXM1 GINS1 GTSE1 HMGA2 KIFC1 MCM3 MCM4 MCM8 MELK NCAPG NCAPG2 NCAPH NEK2 NUF2 PIM2 PRIM1 PRKAR2B PRKCA RPA4 SKA1 SMC1A SPAG5 STMN1
**15/204**	1.903e−06	DNA-REPLICATION	BRCA1 BRCA2 CDC45 CDK2 CHAF1B DTL GINS1 HMGA1 MCM3 MCM4 MCM8 POLQ PRIM1 RFC3 RPA4
**5/17**	4.036e−06	REGULATION-OF-PLATELET-AGGREGATION	ADRB2 LYN PRKCA PRKCD PTGER3
**10/97**	4.105e−06	DNA-DEPENDENT-DNA-REPLICATION	BRCA2 CDC45 GINS1 HMGA1 MCM3 MCM4 POLQ PRIM1 RFC3 RPA4
**11/119**	4.267e−06	KINETOCHORE	AURKB CENPE CENPI CENPM CENPW NEK2 NUF2 SGOL2 SKA1 SMC1A SPAG5
**13/170**	5.487e−06	SISTER-CHROMATID-SEGREGATION	AURKB CENPE CENPI CENPM KIFC1 NCAPG NCAPH NEK2 NUF2 SGOL2 SKA1 SMC1A SPAG5
**16/253**	6.399e−06	CHROMOSOME-SEGREGATION	AURKB BRCA1 BRCA2 CENPE CENPI CENPM CENPW KIFC1 NCAPG NCAPH NEK2 NUF2 SGOL2 SKA1 SMC1A SPAG5
**16/262**	9.929e−06	DNA-CONFORMATION-CHANGE	CDC45 CENPI CENPM CENPW CHAF1B GINS1 HIST1H2BG HIST1H3G HMGA1 HMGA2 HMGB3 MCM3 MCM4 NCAPG NCAPG2 NCAPH
**22/448**	9.939e−06	CELL-DIVISION	AURKB BCL2L1 BRCA2 CDCA3 CDK2 CDK6 CENPE CENPW DIAPH2 FBXO5 HMGA2 KIFC1 NCAPG NCAPG2 NCAPH NEK2 NUF2 SGOL2 SKA1 SMC1A SPAG5 STMN1
**10/107**	9.953e−06	CELL-CYCLE-G1-S-PHASE-TRANSITION	CDC45 CDK2 CDK6 FBXO5 MCM3 MCM4 MCM8 PIM2 PRIM1 RPA4
**33/851**	1.418e−05	CHROMOSOME	AURKB BRCA1 BRCA2 CDC45 CDK2 CENPE CENPI CENPM CENPW CHAF1B DTL GINS1 HIST1H2AE HIST1H2BG HIST1H3G HMGA1 HMGA2 HMGB3 MCM3 MCM4 MKI67 NCAPG NCAPH NEK2 NUF2 POLQ PRIM1 RFC3 RPA4 SGOL2 SKA1 SMC1A SPAG5
**5/24**	2.556e−05	HISTONE-PHOSPHORYLATION	AURKB CDK2 HMGA2 PRKCA PRKCD
**17/322**	3.577e−05	CHROMOSOMAL-REGION	AURKB BRCA2 CDK2 CENPE CENPI CENPM CENPW HIST1H3G MCM3 MCM4 MKI67 NEK2 NUF2 SGOL2 SKA1 SMC1A SPAG5
**35/971**	3.644e−05	CHROMOSOME-ORGANIZATION	ATAD2 AURKB BRCA1 BRCA2 CDC45 CDK2 CENPE CENPI CENPM CENPW CHAF1B GINS1 HIST1H2AE HIST1H2BG HIST1H3G HMGA1 HMGA2 HMGB3 KDM5C KIFC1 MCM3 MCM4 NCAPG NCAPG2 NCAPH NEK2 NUF2 PRIM1 PRKCA PRKCD RFC3 SGOL2 SKA1 SMC1A SPAG5
**47239**	6.739e−05	DNA-REPLICATION-INITIATION	CDC45 MCM3 MCM4 PRIM1 RPA4
**8/87**	7.646e−05	MITOTIC-SISTER-CHROMATID-SEGREGATION	AURKB CENPE KIFC1 NCAPG NCAPH NEK2 SMC1A SPAG5
**14/250**	8.358e−05	CELL-CYCLE-PHASE-TRANSITION	CDC45 CDK2 CDK6 FBXO5 FOXM1 MCM3 MCM4 MCM8 MELK NEK2 PIM2 PRIM1 PRKAR2B RPA4
**20/466**	1.491e−04	ORGANELLE-FISSION	AURKB BRCA2 CDCA3 CDK2 CENPE CENPW FBXO5 HMGA2 KIFC1 MKI67 NCAPG NCAPG2 NCAPH NEK2 NUF2 PIM2 SGOL2 SKA1 SMC1A SPAG5
**11/183**	2.287e−04	DNA-PACKAGING	CENPI CENPM CENPW CHAF1B HIST1H2BG HIST1H3G HMGA1 HMGA2 NCAPG NCAPG2 NCAPH
**16/351**	3.121e−04	MITOTIC-NUCLEAR-DIVISION	AURKB CDCA3 CDK2 CENPE CENPW FBXO5 HMGA2 KIFC1 NCAPG NCAPG2 NCAPH NEK2 NUF2 SKA1 SMC1A SPAG5
**8/109**	3.694e−04	SISTER-CHROMATID-COHESION	AURKB CENPE CENPI CENPM NUF2 SGOL2 SKA1 SMC1A
**3/11**	3.906e−04	INNER-CELL-MASS-CELL-PROLIFERATION	BRCA2 GINS1 NCAPG2
**3/11**	3.906e−04	MCM-COMPLEX	MCM3 MCM4 MCM8

**15/328**	4.458e−04	REGULATION-OF-DNA-METABOLIC-PROCESS	ANXA3 AURKB BRCA1 BRCA2 CDK2 FGF10 FOXM1 HMGA2 NEK2 POLQ PRKCD RAD51AP1 RFC3 SMC1A TOM1L1
**3/12**	5.163e−04	CELL-CYCLE-DNA-REPLICATION	CDC45 GINS1 RPA4
**9/143**	5.424e−04	REGULATION-OF-RESPONSE-TO-DNA-DAMAGE-STIMULUS	BCL2L1 BRCA1 CD74 FGF10 FOXM1 HMGA2 POLQ PRKCD RAD51AP1
**11/213**	8.247e−04	NEGATIVE-REGULATION-OF-CYTOSKELETON-ORGANIZATION	AURKB BRCA1 EZR FBXO5 KIFC1 NEK2 PRKCD SMC1A SPAG5 STMN1 TMOD2
**3/14**	8.394e−04	ATTACHMENT-OF-SPINDLE-MICROTUBULES-TO-KINETOCHORE	AURKB BRCA2 CENPE
**3/14**	8.394e−04	DNA-APURINIC-OR-APYRIMIDINIC-SITE-LYASE-ACTIVITY	HMGA1 HMGA2 NEIL3
**5/49**	8.574e−04	REGULATION-OF-GENE-SILENCING	ATAD2 CDC45 CDK2 HIST1H3G HMGA1
**4/30**	9.106e−04	CHROMOSOME-CONDENSATION	HMGA2 NCAPG NCAPG2 NCAPH
**6/73**	9.535e−04	REGULATION-OF-DNA-REPAIR	BRCA1 FGF10 FOXM1 HMGA2 POLQ RAD51AP1
**24/698**	9.573e−04	CELLULAR-RESPONSE-TO-DNA-DAMAGE-STIMULUS	BCL2L1 BRCA1 BRCA2 CDC45 CDK2 CHAF1B DTL FOXM1 GTSE1 HMGA1 HMGA2 LYN MAP2K6 MCM8 NEIL3 NFATC2 POLQ RAD51AP1 RFC3 RPA4 SMC1A TNFRSF1B UBE2T VAV3
**16/392**	1.020e−03	POSITIVE-REGULATION-OF-LOCOMOTION	ANXA3 CD274 CD74 CXCL5 DOCK5 ENPP2 FGF10 GTSE1 LYN PODXL PRKCA PTGER3 RRAS2 SEMA7A SRPX2 WNT5B
**4/31**	1.035e−03	CELLULAR-SENESCENCE	HMGA1 HMGA2 PRKCD TBX3
**3/15**	1.040e−03	FEMALE-GENITALIA-DEVELOPMENT	AXL FGF10 TBX3
**3/15**	1.040e−03	DNA-DAMAGE-RESPONSE-SIGNAL-TRANSDUCTION-RESULTING-IN-TRANSCRIPTION	BRCA1 BRCA2 FOXM1
**6/75**	1.100e−03	RECOMBINATIONAL-REPAIR	BRCA1 BRCA2 CDC45 MCM8 POLQ RAD51AP1

Supplemental table 3. Gene Set Enrichment analysis (GSEA) and genes upregulated in DM1 derived fibroblasts. *p* –value shows significance of the gene set enrichment in DM1 compared to control fibroblasts.

Count	p-val	GSEA	Genes
**4/84**	1.476e−05	COLLAGEN-TRIMER	COL14A1 COLEC12 CTHRC1 SCARA3
**4/107**	3.838e−05	POTASSIUM-CHANNEL-ACTIVITY	ABCC9 KCNA4 KCND2 KCNE4
**2/10**	9.204e−05	CARDIAC-LEFT-VENTRICLE-MORPHOGENESIS	CPE SFRP2
**3/80**	3.164e−04	VOLTAGE-GATED-POTASSIUM-CHANNEL-ACTIVITY	KCNA4 KCND2 KCNE4
**3/83**	3.528e−04	POTASSIUM-CHANNEL-COMPLEX	ABCC9 KCNA4 KCND2
**5/394**	7.756e−04	EXTRACELLULAR-MATRIX	COL14A1 CTHRC1 EFEMP1 MFAP5 SFRP2
**2/30**	8.777e−04	WNT-PROTEIN-BINDING	CTHRC1 SFRP2
**3/119**	1.013e−03	EXTRACELLULAR-MATRIX-COMPONENT	COL14A1 EFEMP1 MFAP5
**2/34**	1.129e−03	DELAYED-RECTIFIER-POTASSIUM-CHANNEL-ACTIVITY	KCNA4 KCND2
**2/37**	1.337e−03	COLLAGEN-FIBRIL-ORGANIZATION	COL14A1 SFRP2
**2/42**	1.723e−03	SCAVENGER-RECEPTOR-ACTIVITY	COLEC12 SCARA3
**4/325**	2.594e−03	PROTEINACEOUS-EXTRACELLULAR-MATRIX	COL14A1 CTHRC1 EFEMP1 MFAP5
**2/52**	2.635e−03	EPIDERMAL-GROWTH-FACTOR-RECEPTOR-SIGNALING-PATHWAY	CBL EFEMP1
**2/72**	5.009e−03	EXTRACELLULAR-MATRIX-STRUCTURAL-CONSTITUENT	COL14A1 MFAP5
**2/78**	5.860e−03	REGULATION-OF-JUN-KINASE-ACTIVITY	SFRP2 TNFRSF11A
**3/228**	6.438e−03	SENSORY-ORGAN-MORPHOGENESIS	CTHRC1 EFEMP1 NR4A3
**2/83**	6.617e−03	PLASMA-MEMBRANE-RAFT	CBL KCND2
**3/233**	6.837e−03	PROTEIN-HOMOOLIGOMERIZATION	COLEC12 KCNA4 KCND2
**2/88**	7.417e−03	INNER-EAR-MORPHOGENESIS	CTHRC1 NR4A3
**7/1227**	8.881e−03	RECEPTOR-ACTIVITY	ABCC9 COLEC12 EFEMP1 NR4A3 SCARA3 SFRP2 TNFRSF11A
**4/470**	9.528e−03	PLASMA-MEMBRANE-PROTEIN-COMPLEX	ABCC9 CBL KCNA4 KCND2
**4/477**	1.002e−02	INORGANIC-CATION-TRANSMEMBRANE-TRANSPORTER-ACTIVITY	ABCC9 KCNA4 KCND2 KCNE4
**3/269**	1.015e−02	EMBRYONIC-ORGAN-MORPHOGENESIS	CTHRC1 EFEMP1 NR4A3
**2/105**	1.045e−02	ION-CHANNEL-BINDING	ABCC9 KCNE4
**2/108**	1.104e−02	REGULATION-OF-ESTABLISHMENT-OF-PLANAR-POLARITY	CTHRC1 SFRP2
**2/108**	1.104e−02	EAR-MORPHOGENESIS	CTHRC1 NR4A3
**6/1000**	1.111e−02	SIGNALING-RECEPTOR-ACTIVITY	ABCC9 COLEC12 EFEMP1 NR4A3 SFRP2 TNFRSF11A
**2/109**	1.123e−02	REGULATION-OF-OSTEOBLAST-DIFFERENTIATION	CTHRC1 SFRP2

**3/281**	1.143e−02	GATED-CHANNEL-ACTIVITY	KCNA4 KCND2 KCNE4
**3/286**	1.199e−02	EXTRACELLULAR-STRUCTURE-ORGANIZATION	COL14A1 MFAP5 SFRP2
**3/286**	1.199e−02	TRANSPORTER-COMPLEX	ABCC9 KCNA4 KCND2
**4/513**	1.283e−02	INORGANIC-ION-TRANSMEMBRANE-TRANSPORT	ABCC9 KCNA4 KCND2 KCNE4
**5/763**	1.291e−02	POSITIVE-REGULATION-OF-CELL-PROLIFERATION	CTHRC1 EFEMP1 NR4A3 SFRP2 TNFRSF11A
**4/518**	1.326e−02	EMBRYONIC-MORPHOGENESIS	CTHRC1 EFEMP1 NR4A3 SFRP2
**7/1328**	1.327e−02	SIGNAL-TRANSDUCER-ACTIVITY	ABCC9 CBL COLEC12 EFEMP1 NR4A3 SFRP2 TNFRSF11A
**4/520**	1.343e−02	METAL-ION-TRANSPORT	ABCC9 KCNA4 KCND2 KCNE4
**2/126**	1.485e−02	TUBE-FORMATION	CTHRC1 SFRP2
**8/1674**	1.537e−02	REGULATION-OF-TRANSPORT	CBL KCNA4 KCND2 KCNE4 NR4A3 SFRP2 SYT14 TNFRSF11A
**3/314**	1.541e−02	TUBE-MORPHOGENESIS	CTHRC1 NR4A3 SFRP2
**2/131**	1.600e−02	CIRCADIAN-RHYTHM	KCND2 TNFRSF11A

Supplemental table 4. Clinical characteristics of the individuals from whom PBMCs were isolated and used in this study.

Code controls	Gender	Age at blood collection
F-1	Female	40
F-2	Female	42
F-3	Female	38
F-4	Female	44
F-5	Female	59
F-6	Female	43
F-7	Female	51
F-8	Female	57
F-9	Female	55
F-10	Female	44
M-1	Male	40
M-2	Male	53
M-3	Male	42
M-4	Male	45
M-5	Male	43
M-6	Male	42
M-7	Male	53
M-8	Male	41
M-9	Male	46
M-10	Male	31
M-11	Male	34
M-12	Male	54

Code DM1	Gender	Age at blood collection	CTG	MIRS
DM1-F-1	Female	46	667	3
DM1-F-2	Female	47	1167	3
DM1-F-3	Female	35	1167	4
DM1-F-4	Female	53	1667	
DM1-F-5	Female	41	667	3
DM1-F-6	Female	31	500	2
DM1-F-7	Female	37	1333	3
DM1-F-8	Female	18	667	1
DM1-F-9	Female	36	1000	3
DM1-F-10	Female	44	1000	4
DM1-F-11	Female	56	667	4
DM1-F-12	Female	29	467	2
DM1-F-13	Female	50	600	3
DM1-F-14	Female	37	1400	3
DM1-F-15	Female	45	667	3
DM1-F-16	Female	32	500	
DM1-M-1	Male	27	500	
DM1-M-2	Male	31	333	2
DM1-M-3	Male	26	1000	
DM1-M-4	Male	24	467	3
DM1-M-5	Male	51	1333	4
DM1-M-6	Male	35	1000	3
DM1-M-7	Male	50	667	3
DM1-M-8	Male	39	1333	4
DM1-M-9	Male	37	1000	2
DM1-M-10	Male	37	1000	
DM1-M-11	Male	36	400	3
DM1-M-12	Male	35	667	3
DM1-M-13	Male	37	1000	2

DM1-M-14	Male	58	833	3
DM1-M-15	Male	56	1000	3
DM1-M-16	Male	49	667	
DM1-M-17	Male	53	800	4
DM1-M-18	Male	42	667	4
DM1-M-19	Male	41	500	3
DM1-M-20	Male	37	1000	3
DM1-M-21	Male	34	667	3

Code DM1 Cancer	Gender	Age at blood collection	CTG	MIRS	Age of cancer diagnosis
DM1-F-C1	Female	65	1500	4	52
DM1-F-C2	Female	42	833	3	43
DM1-F-C3	Female	39	833		
DM1-F-C4	Female	61	1167	4	39
DM1-F-C5	Female	41	1000	4	35
DM1-F-C6	Female	38	1000	4	37
DM1-F-C7	Female	41	500	4	38
DM1-F-C8	Female	65	333	4	65
DM1-F-C9	Female	51	1500	4	41 and 51
DM1-F-C11	Female	34	1650	4	17
DM1-F-C12	Female	48	1333	3	45
DM1-F-C13	Female	48	1333	2	43 and 47
DM1-M-C1	Male	48	333	3	31
DM1-M-C2	Male	52	1000	3	49
DM1-M-C3	Male	56	333	3	52
DM1-M-C4	Male	57	1333	3	50
DM1-M-C5	Male	55	267		
DM1-M-C6	Male	54	333	4	51

Supplemental Table 5: List of primers used in the manuscript.

HUMAN		
Gene	Forward Sequence (5' to 3')	Reverse Sequence (5' to 3')
GAPDH	ATGGGGAAGGTGAAGGTCGG	GACGGTGCCATGGAATTTGC
DMPK	GGCCAGGTGTATGCCATGAA	CCGCCCACGTAATACTCCA
MBNL1	GCTGTTAGTGTCACACCAATTCG	AGGCGATTACTCGTCCATTTTC
SIX5	GCTGCCTTCGGCCACT	GCCACACCCGTCACGAT
MBNL2 Ex7	ACAAGTGACAACACCGTAACCG	TTTGGTAAAGGATGAAGAGCACC
CDK2	GTACCTCCCCTGGATGAAGAT	CGAAATCCGCTTGTTAGGGTC
HMGA2	ACCCAGGGGAAGACCCAAA	CCTCTTGGCCGTTTTTCTCCA
CENPA	CCGCCTGGCAAGAGAAATATG	GCCAGTTGCACATCCTTTGG
CENPE	GAAGTCAACAGGCCCAAGATAC	CACCTCCACAAGTTAAGGGTTT
CENPI	ACACGGATATTGAAGTGCATGAT	TACCTTGGTGCTACCAGAACA
CDC45	GTGATTTGGCGGGAGTCTTG	CGAAGAGAAGGACCCTCTGG
MCM2	ATCTACGCCAAGGAGAGGGT	GCTGCCTGTCGCCATAGATT
GINS1	TGTATGACCGCTTGCTTCGG	GCAGCCATGTGAAATCGTAATG
AURKB	CATGAGCCGCTCCAATGTC	TGCTATTCTCCATCACCTTCTGG
BRCA1	CAGAAGAAAGGGCCTTCACA	TGTGTCAAGCTGAAAAGCACA
BRCA2	CACCTCTGGAGCGGACTTAT	ATCTGCTTTGTTGCAGCGTG
RAD50	AGGAAAGGCTTTGAAGCAAAA	GTCTGACGTACCTGCCGAAG
RAD51	AGCTCAAGTGGATGGAGCAG	TTCTGGTTTCCCCTCTTCCT
RAD51AP	ATGACAAGCTCTACCAGAGAGAC	CACATTAGTGGTGACTGTTGGAA
FANCA	TGTGGCATCTTCACGTACAAGG	CCTGAAGAGCCACGATCCC
POLQ	ACTTTTGCTGACCAAGATTTGCT	ACTCATGCCAACGATTTGCAC
ABCC9	TTACATTCGCTCTCCTGTTTGTG	GGTGGAGGTGCCTTGATTCC
KCND2	GGGTTTTTCATTGCCGTCTCT	CACAGCATACCGCTCTCCA
SFRP2	CTGGCCCGACATGCTTGAG	GCTTCACATACCTTTGGAGCTT
p16INK4a	GGGGGCACCAGAGGCAGT	GGTTGTGGCGGGGGCAGTT
p14ARF	CCCTCGTGCTGATGCTACTG	CATCATGACCTGGTCTTCTAGGAA
p27KIP1	GCAACCGACGATTCTTCTAC	CTTCTGAGGCCAGGCTTCTT
p21CIP1	GACACCACTGGAGGGTGACT	CAGGTCCACATGGTCTTCCT
BMI1	GGAGACCAGCAAGTATTGTCCTATTT	CATTGCTGCTGCTGGGCATCGTAAG

IL6	CCAGGAGCCCAGCTATGAAC	CCCAGGGAGAAGGCAACTG
TNFA	CCCAGGCAGTCAGATCATCTTC	AGCTGCCCCTCAGCTTGA
CCL5	CTCGCTGTCATCCTCATTGCT	TGTGGTGTCCGAGGAATATGG
CASP5	TTCAACACCACATAACGTGTCC	GTCAAGGTTGCTCGTTCTATGG
MOUSE		
Gene	Forward Sequence (5' to 3')	Reverse Sequence (5' to 3')
Actin	GGCACCACACCTTCTACAATG	GTGGTGGTGAAGCTGTAGCC
Cdk2	CCTGCTTATCAATGCAGAGGG	GTGCTGGGTACACACTAGGTG
Hmga2	AGACCCAGAGGAAGACCCAAAG	TTCAGTCTCCTGAGCAGGCTTC
Cenpa	CTCCAGTGTAGGCTCTCAGAC	CTGAAAGGCTTCTTCCTGAACA
Cenpe	CTCCGCCATACAAGGCTACAA	CCCTGGGTATAACTCCCAAACAA
Cenpi	AGGGTTACTAGAAACTCCCAGC	GCGTGTAGAATCTTCCACTGAA
Cdc45	TAGTGGAGCGAAACAGGAAGA	GGCCGACGATGTCCCATAAT
Mcm2	TATGACGGCTCGCTTAACACC	TTCATGCCCATAGATGGAGGG
Gins1	ATGTTCTGCGAAAAAGCTATGGA	TCACATCAGACTGGTTTTGTTCA
AurkB	CAACTTTGAGATTGGGCGTCC	GGCGAAGCTGGTGCTCTAC
Brca1	CTGCCGTCCAAATTCAAGAAGT	CTTGTGCTTCCCTGTAGGCT
Brca2	TGGTAGATGTTGCTAGTCCGC	ACCACTGGCTTTTCTCGTTGT
Rad50		
Rad51	AAGTTTTGGTCCACAGCCTATTT	CGGTGCATAAGCAACAGCC
Fanca	AGAGCAGATAAGATTCCCTCCTC	GGGTCGGGTCTGTCATTAAAG
PolQ	CAAGGTTTCATTCGGGTCTTGG	CGAGCAGGAAGATTCACTCCAG
Kcnd2	TCAGGACGCTCTGATAGTGCT	TCTGGGTATCGTTCCAGGGTG
Ki-67	ATCATTGACCGCTCCTTTAGGT	GCTCGCCTTGATGGTTCCT
p16Ink4	CCCAACGCCCCGAACT	GCAGAAGAGCTGCTACGTGAA
p19Arf	GCCGCACCGGAATCCT	TTGAGCAGAAGAGCTGCTACGT
p27Kip1	ATTGGGTCTCAGGCAAACTCT	GTTCTGTTGGCCCTTTTGTTT
p21Cip1	GTGGGTCTGACTCCAGCCC	CCTTCTCGTGAGACGCTTAC
Bmi1	ACGTCATGTATGAAGAGGAACCT	TGGCCGAACTCTGTATTTCAAAG

FLY		
Gene	Forward Sequence (5' to 3')	Reverse Sequence (5' to 3')
Rp49	ATCGGTTACGGATCGAACAA	GACAATCTCCTTGCGCTTCT
Mbl	TTGAATCAAAATTATAGCCCAAGCT	CGATTTTGCTCGTTAGCGTTT
Cid	ACAACTCAAAGTCGCCGAAC	CGATTGTTGTCCTGAAGCGT
Cana	CTGCAACAGTTCACCAAGCT	GCTTTGATCCTGTTCGGCAT
String	GAAAACAACTGCAGCATGGAT	CGACAGCTCCTCCTGGTC
Dacapo	GTCAGCTTCCAGGAGTCGAG	CCAAAGTTCTCCCGTTCTGA
Psc	CCAAGCAGAACAGTGTCACC	GCTCGACGATTTGGGACTTC
